# Cobamide excretion allows *Salmonella* to transition from its anaerobic to aerobic lifestyle

**DOI:** 10.1128/spectrum.01363-26

**Published:** 2026-07-24

**Authors:** Lahiru Malalasekara, Jorge C. Escalante-Semerena

**Affiliations:** 1 Department of Microbiology, University of Georgia, Athens, Georgia, USA; Reichman University, Herzeliya, Israel

**Keywords:** corrinoid excretion, *Salmonella *metabolism, cobamide-dependent growth, *Salmonella *lifestyle transition

## Abstract

Cobamides (Cbas) are structurally complex biomolecules used by cells of all domains of life. However, *de novo* Cba biosynthesis occurs only in some bacteria and archaea. The uptake, salvage, and remodeling of Cbas and their precursors have been well studied in several prokaryotes, but our understanding of the mechanism(s) of Cba release into the environment is limited. In this study, we quantitatively demonstrate Cba excretion by *Salmonella enterica* subsp. *enterica* sv. Typhimurium strain LT2 (hereafter *S*. Typhimurium). We performed experiments in liquid and solid media using strains that produced Cbas from a precursor that can be converted to Cbas in the presence of oxygen. We show that under anaerobic conditions, *S*. Typhimurium synthesizes Cbas *de novo* and excretes this valuable cofactor into the medium. To do this, *S*. Typhimurium requires ≥24 h of incubation under anaerobic conditions to form microcolonies that can continue synthesizing and excreting Cbas when shifted to aerobic growth conditions. We propose that *de novo* Cba biosynthesis and excretion may be necessary for *S*. Typhimurium to transition from an anaerobic to an aerobic environment, and that Cba excretion may allow growth of Cba-requiring members of polymicrobial communities.

**IMPORTANCE:**

This work is important for several reasons. First, this is the first evidence that *Salmonella enterica* subsp. *enterica* sv. Typhimurium strain LT2 excretes cobamides, a valuable nutrient for all forms of life, including humans. Second, although *de novo* cobamide biosynthesis in *S*. Typhimurium is blocked by oxygen, we show that once a microcolony of *S*. Typhimurium is formed under anaerobic conditions, exposure to air does not block cobamide biosynthesis, and robust colony growth can proceed. Hence, the formation of microcolonies under anaerobic conditions may be a critical strategy for *S*. Typhimurium survival in its free-living lifestyle. Third, in an environment where *S*. Typhimurium is part of a polymicrobial community, this pathogen may allow growth of other microbes that require cobamides to survive, as it may occur in polymicrobial biofilms. Finally, this work provides the groundwork for advancing our understanding of why *S*. Typhimurium excretes such a valuable cofactor and how excretion is accomplished.

## INTRODUCTION

Corrinoids are a group of cyclic biomolecules composed of four reduced pyrrole rings linked at their C∝ position, with three of them separated by a one-carbon unit and two pyrroles being directly linked at their C∝ carbons. The resulting ring structure is known as the corrin ring because it is the core structure of all corrinoids, the best known of which is vitamin B_12_ (aka. cyanocobalamin [CNCbl]).

**Fig 1:**
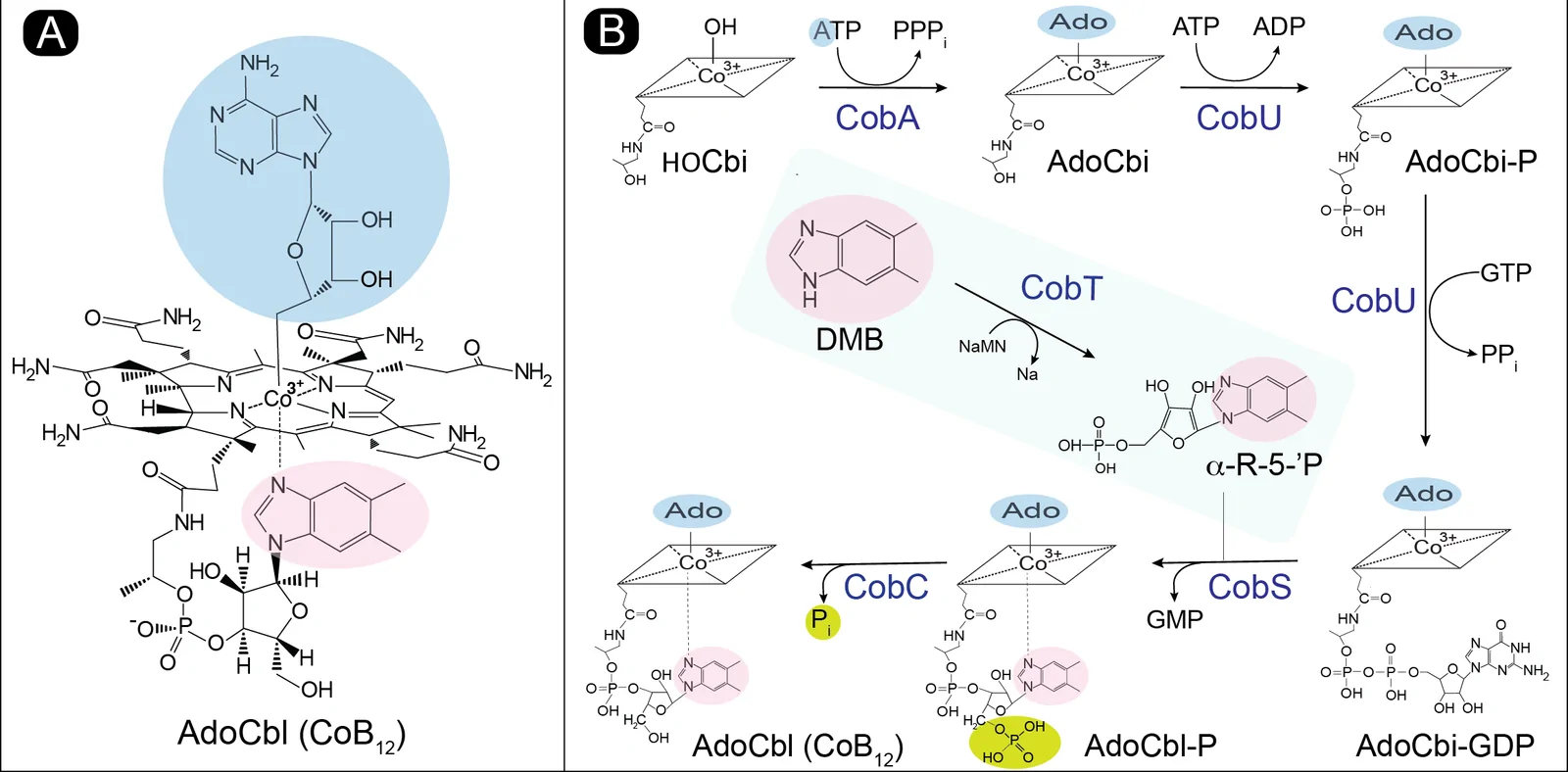
(**A**) Chemical structure of coenzyme B_12_ (CoB_12_, aka adenosylcobalamin). The upper axial ligand (5′-deoxyadenosine) and lower ligand nucleobase (5,6-dimethylbenzimidazole [DMB]) are highlighted in blue and pink, respectively. (**B**) Selected steps of cobamide biosynthesis in *Salmonella* relevant to this work. The upper ligand is attached to the cobalt ion of the corrin ring by the housekeeping ATP:Co(I)rrinoid adenosyltransferase (CobA, EC 2.5.1.17) upon uptake by the cell. Adenosylcobinamide (AdoCbi) is activated to adenosylcobinamide-GDP (AdoCbi-GDP) by the bifunctional NTP:AdoCbi kinase (CobU, EC 2.7.7.62), GTP:AdoCbi-P guanylyltransferase (CobU, EC 2.7.1.156) that yields AdoCbi-GDP via AdoCbi-P. The lower ligand DMB is activated into ∝-ribazole-5′-phosphate by the NaMN:DMB phosphoribosyl transferase (CobT, EC 2.4.2.21). The activated corrin ring and activated nucleobase are then condensed into adenosylcobalamin-5′-phosphate (AdoCbl-P) by AdoCbl-P synthase (CobS, EC 2.7.8.26) in the penultimate step of CoB_12_ biosynthesis. AdoCbl-P is finally dephosphorylated by the AdoCbl-P phosphatase (CobC, EC 3.1.3.73) to yield CoB_12_. The rhomboid structure with the Co^3+^ ion in the middle is a cartoon of the complex cyclic tetrapyrrole ring (corrin ring) shown in panel **A**.

As seen in Fig. 1A, a complete corrinoid (aka cobamide [Cba]) contains a cobalt ion held by equatorial interactions with the pyrrolic nitrogens and by upper (*Co*ß) and lower (*Co*∝) axial ligands. The structural diversity among Cbas is attributed to the differences in the axial ligands. For example, cobalamin (Cbl) is the Cba in which the nucleobase 5,6-dimethylbenzimidazole (DMB) is the lower ligand, while in pseudo-cobalamin (psCbl), adenine is the nucleobase that serves as the lower ligand (1). The diversity in upper axial ligands is best illustrated by the cyano group of vitamin B_12_ and the 5′-deoxyadenosine group of adenosylcobalamin, a biologically active form of the vitamin (Fig. 1A).

Cbas are used in nature to perform diverse chemical reactions, including (but not limited to) methyl group transfers, reductive dehalogenases, carbon skeleton rearrangements, elimination reactions, and ribonucleotide reductions (2). Cbas are also used to control gene expression as either a photosensor of a transcription factor or by binding to riboswitches and occluding ribosome-binding sites needed for mRNA translation (3, 4).

Cba assembly is catalyzed by one of the largest metabolic pathways in nature, involving the function of ~30 genes (5). Only a limited genera of bacteria and archaea synthesize Cbas *de novo*. However, cells from all domains of life, including human cells, require Cbas. Although higher plants are not known to use Cbas, ~50% of algae studied use Cbas (6, 7). Analysis of bacterial genomes revealed that most of them (~86%) contained at least one gene encoding a Cba-dependent enzyme and <40% had the genetic capacity to synthesize Cba *de novo* (8, 9). Notably, *de novo* Cba biosynthesis is conditional in some organisms. For instance, *S*. Typhimurium can only *de novo* synthesize Cbas under anaerobic conditions (10). This is due to oxygen-labile steps and the limited expression of genes involved in the assembly of the corrin ring (11, 12).

Although *de novo* Cba biosynthesis is limited to some prokaryotes, its use is widespread. Hence, it is likely that the availability of Cbas and their precursors has a profound impact on members of the biosphere (13, 14). The use and impact of Cbas produced in microbial communities have been studied. Laboratory co-cultures of bacteria and algae are an example of where both species benefit from each other. Bacterial Cba producers provide Cbas needed by algae, and bacteria benefit from nutrients procured by algae (15–17). However, our understanding of how Cbas are released into the environment by Cba-producing microorganisms is limited.

*S*. Typhimurium has been used as a model organism to study *de novo* Cba biosynthesis, precursor salvaging, and transport of corrinoids for decades (10, 18–20). In this study, we show that *S*. Typhimurium can excrete Cbas, and we suggest that this bacterium may benefit from Cba excretion by supplying Cbas to cells growing in the most oxygen-exposed layers of colonies where *de novo* Cba synthesis does not happen. This study serves as the groundwork needed for the characterization of the mechanism of Cba excretion in *S*. Typhimurium and probably other Cba-producing prokaryotes.

## RESULTS

### *S*. Typhimurium converts exogenous cobinamide into cobamides, some of which are excreted

Questions regarding the excretion of Cbas by *S*. Typhimurium arose during studies aimed at identifying corrinoids present in spent minimal media of *S*. Typhimurium cultures grown under conditions that demanded the conversion of incomplete corrinoids into Cbas. *S*. Typhimurium synthesizes the corrin macrocycle *de novo* only under anaerobic conditions but can convert precursors with a complete corrin ring into Cbas under aerobic conditions (10). We describe below results from experiments performed under aerobic conditions using glycerol minimal medium supplemented with dicyanocobinamide [(CN)_2_Cbi] (an incomplete Cba).

#### Analysis of extracellular corrinoids

To analyze the extracellular corrinoids, we removed cells from the medium by centrifugation (6,000 × *g*) followed by filtration through a 0.22-μm filter (Avantor). Corrinoids in the supernatant were partially purified using a SepPak C18 cartridge (Waters) as described in Materials and Methods. We used bioassays to differentiate between incomplete corrinoids and Cbas present in spent medium. To do this, we used *S*. Typhimurium strains that harbored a chromosomal null allele of the *metE* gene encoding the Cba-independent methionine synthase enzyme but retained a wild-type allele of the *metH* gene encoding the Cba-dependent L-methionine synthase (MetH) isozyme. Strains with the abovementioned genetic background were forced to use the MetH methionine synthase to methylate homocysteine to yield methionine. The presence of either methionine, cobinamide, or Cba in the supernatant allowed the Δ*metE metH^+^* to grow, whereas a Δ*metE* Δ*metH* strain grew only if methionine was present in spent medium. We used two additional strains that carried chromosomal deletions of either the *cobS* gene, which encodes the cobalamin-5′-phosphate synthase (21, 22), or a deletion of the *cobA* gene, which encodes the housekeeping corrinoid adenosyltransferase CobA enzyme (23, 24). The roles of the CobA and CobS enzymes in Cba biosynthesis are illustrated in Fig. 1B. Strains devoid of either CobS or CobA cannot convert the precursor cobinamide to Cba and hence cannot grow. We used Δ*cobA* and Δ*cobS* strains to detect the presence of Cbas in samples of interest.

**Fig 2:**
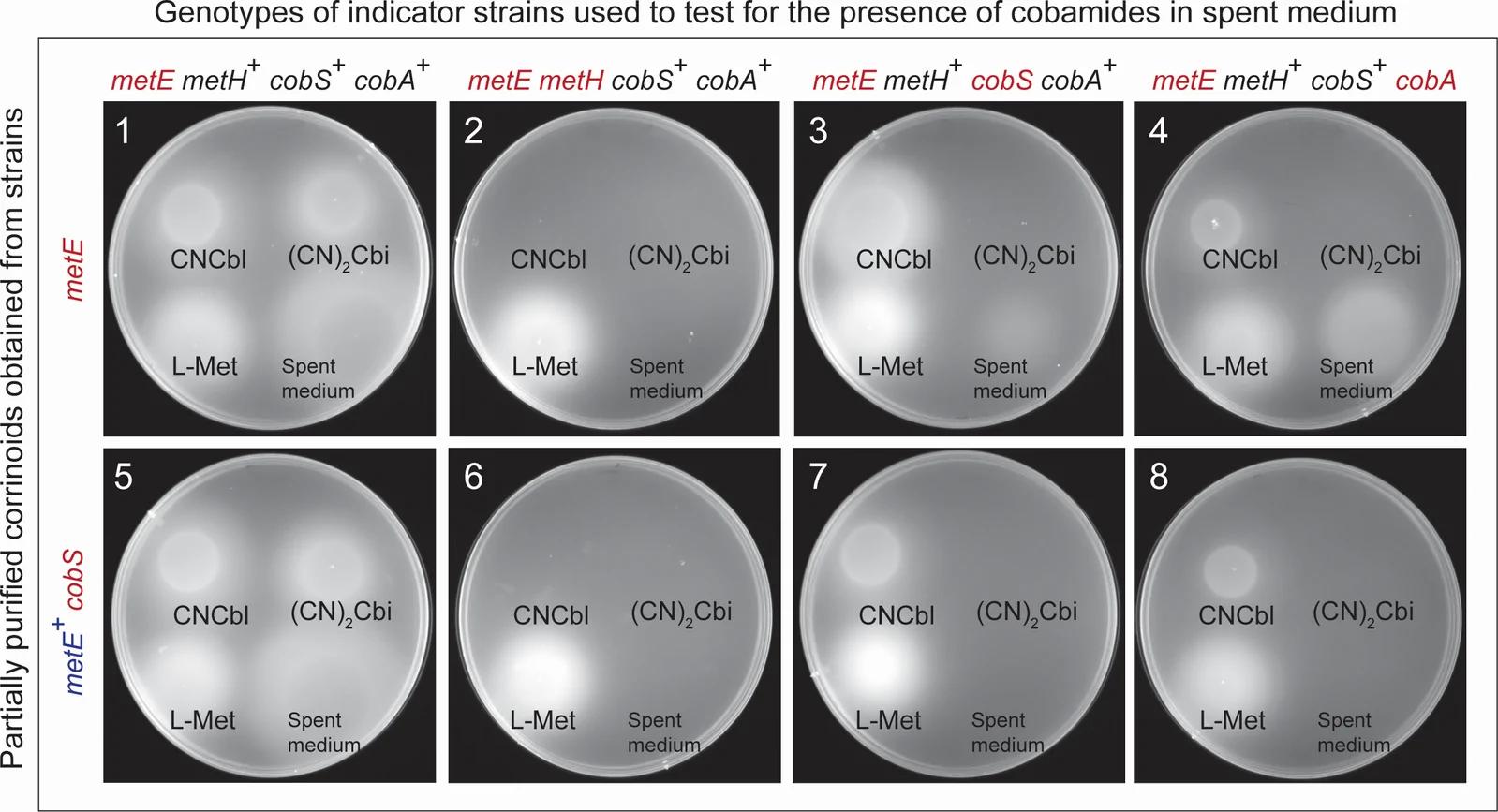
Bioassay for cobamides excreted by *S*. typhimurium. Details of the procedures used in the performance of these experiments are described under Materials and Methods. Strains JE7088 (Δ*metE*) and JE27521 (*metE*^+^ Δ*cobS*) were grown in NCE minimal medium (5 mL) supplemented with glycerol (22 mM) and (CN)_2_Cbi (50 μM). The cell-free filtrate was prepared, and corrinoids were partially purified. Extracted corrinoids were dissolved in sterile water (200 μL), and samples (10 μL) were spotted (location indicated as “spent medium”) on plates seeded with indicator strains whose genotypes are shown on top of each column. Indicator strains were suspended in 0.8% (wt/vol) agar and poured onto the surface of the abovementioned plate. A sample (3 μL) of CNCbl (5 μM), (CN)_2_Cbi (5 μM), or L-methionine (L-Met, 50 mM) was applied on each plate as controls. Panels are identified by numbers. Null alleles are shown in red font. The experiment was repeated three times with a representative result shown.

Unexpectedly, we found Cbas in spent medium, as indicated by the growth of the Δ*cobS* and Δ*cobA* strains in medium containing partially purified spent medium from cultures of a Δ*metE* strain (Fig. 2, panels 3 and 4). To rule out the presence of Cbas in the stock solution of (CN)_2_Cbi used in the previous experiments, we used a *metE*^+^ Δ*cobS* strain that grew because it used the MetE enzyme to make methionine but could not convert (CN)_2_Cbi to Cbas due to the absence of CobS. As expected, the *metE*^+^ Δ*cobS* strain did not convert exogenous (CN)_2_Cbi to Cbas, and the spent medium of this culture did not support growth of the Δ*metE* Δ*cobS* (Fig. 2, panel 7) or the Δ*metE* Δ*cobA* strains (Fig. 2, panel 8). These results were consistent with the idea that strains with functional late steps of Cba biosynthesis were the source of Cbas present in spent medium (Fig. 2, panels 3 and 4).

### Extracellular Cbas can also be detected in liquid co-cultures

**Fig 3:**
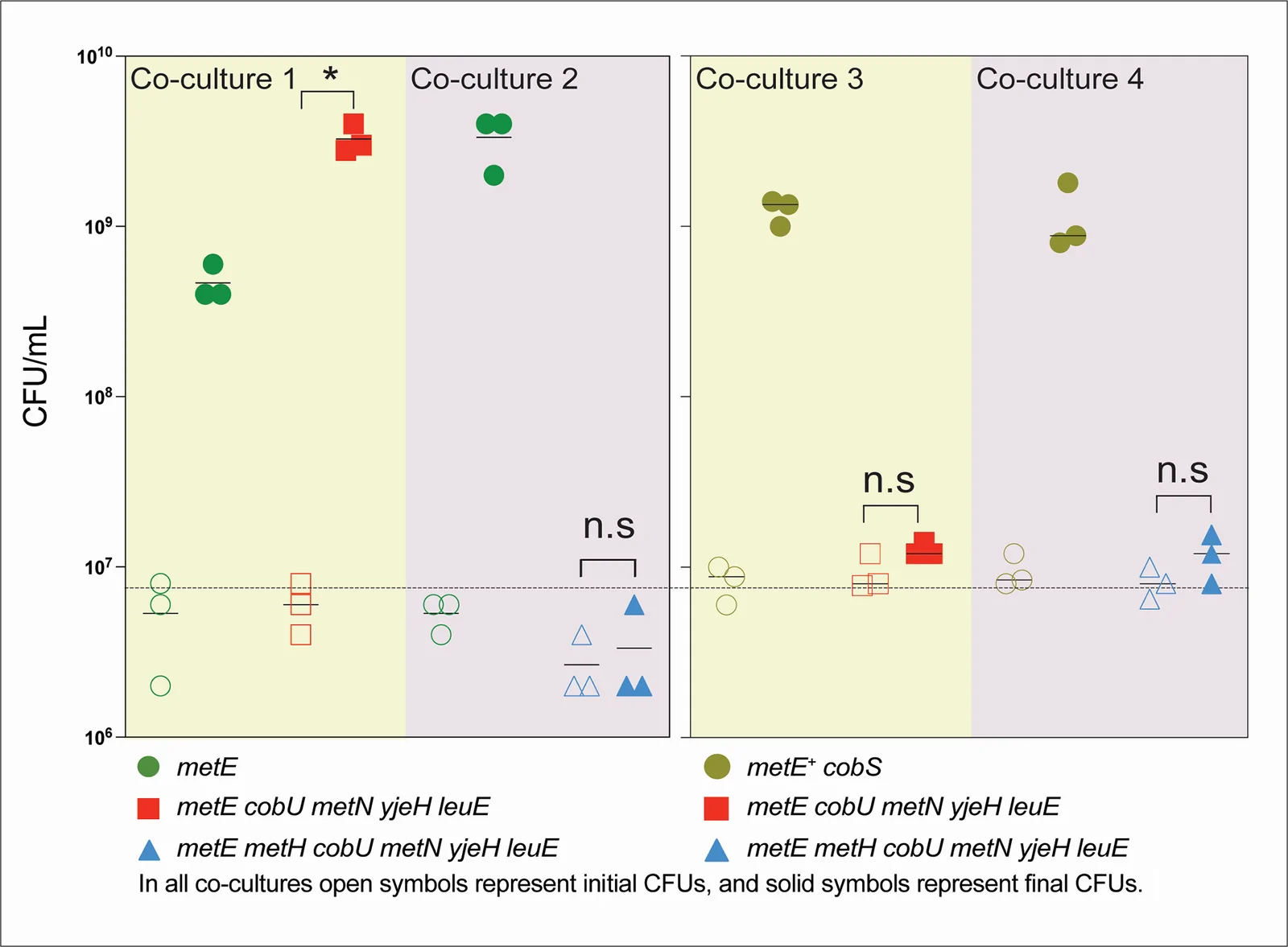
Evidence that Cbas are excreted by cultures grown in liquid medium. Four different co-cultures were grown (1–4) in NCE minimal medium (5 mL) supplemented with glycerol (22 mM), (CN)_2_Cbi (10 nM), and DMB (300 mM). Viable cell counts of each strain were obtained using their unique antibiotic markers at two different time points of growth: initial and early stationary phase (8 h post-inoculation). The experiment was repeated three times with three technical replicates, with a representative result shown. A paired Student’s *t*-test was performed to determine statistical significance (ns, not significant; *P < 0.05).

To verify the presence of extracellular Cbas in a liquid culture, we co-cultured a Cba-producing strain with a Cba auxotroph. We used different antibiotic-resistant markers to identify strains being co-cultured. Co-culture 1 contained the Cba-producing JE7087 strain (chloramphenicol resistant) and the Cba auxotroph JE27701 strain (kanamycin resistant [Km^R^]). In co-culture 2, we substituted the methionine auxotroph JE27702 strain (Km^R^) for the Cba auxotroph JE27701 strain. To quantify the number of viable cells per unit volume (i.e., colony-forming unit [CFU] per milliliter) of each strain, samples from a co-culture under study were diluted 10× and spotted on rich medium plates containing one antibiotic to which one of the strains in the co-culture was resistant. All strains carried a null *metE* allele, making them either methionine or Cba auxotrophs. To block the conversion of (CN)_2_Cbi to Cba, we disrupted the *cobU* gene (see Fig. 1B), yielding the Cba auxotroph. In co-culture 1, we supplemented the medium with (CN)_2_Cbi to allow the Δ*metE* (JE7087) strain to synthesize Cbas and grow (Fig. 3, co-culture 1, green filled circles). We noticed that when the co-culture reached an early stationary phase, the Cba auxotroph (strain JE27701) had outcompeted the Cba producer (strain JE7807; Fig. 3, co-culture 1, red vs green solid symbols). Again, under the experimental conditions used for co-culture 1, either a Cba or methionine would allow the Cba auxotroph (strain JE27701) to grow. To minimize the uptake of any residual methionine from the medium, we introduced mutations in known and predicted methionine uptake systems into the Cba auxotroph (JE27701; see Table 2). To rule out the possibility that growth was supported by exogenous methionine, we introduced a null allele of the *metH* gene into strain JE27701, resulting in strain JE27702 (see Table 2). In the absence of MetH, cells could not methylate homocysteine to yield methionine. In the co-culture of JE27702 (Δ*metH cobU metN yjeH leuE*) and JE7087 (Δ*metE*), strain JE27702 failed to grow, indicating that the medium in the co-culture either lacked or contained insufficient methionine to support growth (Fig. 3, co-culture 2, blue vs green solid symbols). Ectopic expression of *metH* was sufficient to rescue the growth of strain JE27702 in co-culture (Fig. S1A). To determine whether the Cbas that allowed the Cba auxotroph to grow were synthesized by the Cba-producing strain in the co-culture (JE7087), we grew a co-culture of the *metE*^+^ Δ*cobS* strain (JE27521) with the *cobU* strain (JE27701), which is a Cba auxotroph (Fig. 1B). As expected, in the presence of a non-Cba-producing strain (e.g*.,* JE27521, and Δ*cobS*), the Cba auxotroph (JE27701, *cobU metN yjeH leuE*) failed to grow in co-culture (Fig. 3, co-culture 3, red filled squares). Strain JE27701 carried an insertion in the *cobU* gene (Fig. S1C). The lack of growth of strain JE27701 co-cultured with JE27521 was corrected by introducing a plasmid expressing the *cobUST* genes (Fig. S1B). This result confirmed that the inability to convert (CN)_2_Cbi to Cba was the sole reason for the lack of growth of strain JE27701 in the co-culture.

### Visualizing Cba feeding

**Fig 4:**
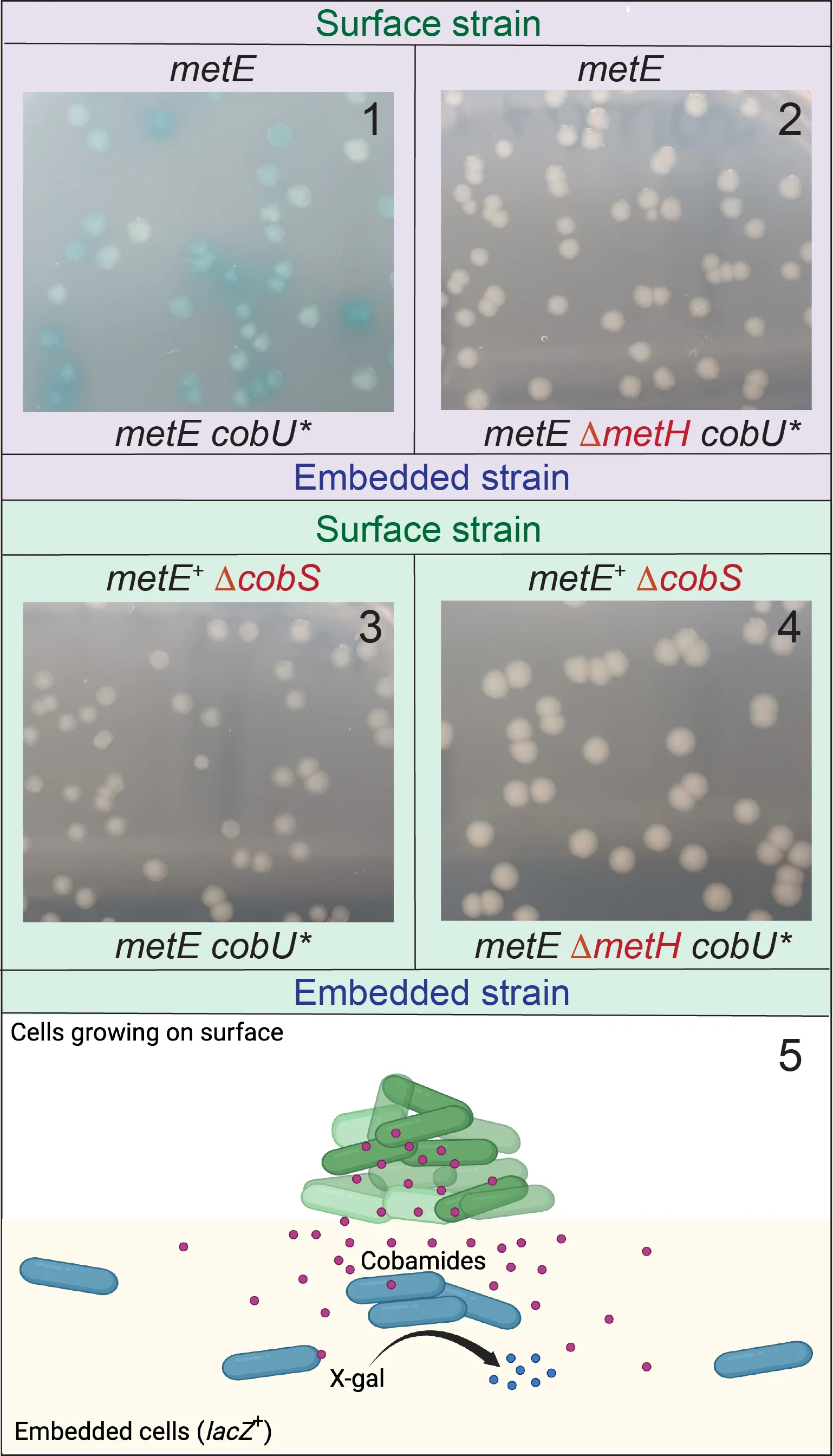
Use of β-galactosidase activity as a reporter of Cba excretion. Strain genotypes describing a *cobU* mutation refer to a *cobU-lacZ^+^* transcriptional fusion (11). Cells from overnight cultures (20 h, in LB) of these strains (JE27701, Δ*metE cobU-lacZ^+^*; JE27702, Δ*metE* Δ*metH cobU-lacZ^+^*) were washed twice with a NaCl solution (0.9% wt/vol), and a sample (100 μL) was embedded into NCE agar (25 mL, 1.5% wt/vol) supplemented with glycerol (22 mM), CN_2_Cbi (10 nM), DMB (300 μM), and 5-bromo-4-chloro-3-indolyl-β-D-galacto-pyranoside (X-gal, 40 μg/mL). After the agar solidified, ~100 to 200 CFUs of either strain JE7087 (Δ*metE*) or JE27521 (*metE^+^* Δ*cobS*) were spread onto the surface. Plates were incubated at 37°C for 48 h. Panels are identified by numbers. Experiments were repeated three times with representative results shown. The asterisk on the genotype of strains JE27701 and JE27702 indicates the presence of chromosomal null alleles of Δ*metN*, Δ*yjeH*, and Δ*leuE* in the strains. The illustration was created with BioRender.com.

We performed four experiments to visualize Cba feeding. First, we embedded a Cba auxotroph (JE27701, *metE cobU-lacZ^+^*) in minimal medium agar plates containing (CN)_2_Cbi plus the chromogen 5-bromo-4-chloro-3-indolyl-β-D-galactopyranoside (X-gal). A Cba-producing strain (JE7087, *metE cob^+^*) was grown in lysogeny broth (LB) to full density (~20 h of incubation at 37°C with shaking), diluted to ~10^3^ CFU/mL, and a sample (0.1 mL) was spread onto X-gal plates. Strain JE7087 had all the enzymes needed to convert (CN)_2_Cbi to a Cba, allowing the Cba-dependent MetH enzyme to synthesize methionine needed for growth. Since the Cba auxotroph embedded in the agar carried a *cobU-lacZ^+^* transcriptional fusion (+), metabolically active cells of the Cba auxotroph were identified by the blue color around the Cba-producing strain (JE7087), which excreted Cbas that cells of the Cba-auxotroph located under colonies of strain JE7087 used to grow. The blue color resulted from the cleavage of the X-gal chromophore by β-galactosidase produced by the Cba auxotroph (Fig. 4, panel 1). As a control for this experiment, a culture of the strain carrying the *cobU-lacZ^+^* transcriptional fusion (JE27701) was grown overnight in LB medium and diluted 10^6^-fold, and a sample (0.3 mL) was embedded into minimal glycerol medium supplemented with X-gal and either CNCbl or L-methionine (Fig. S2). The results of this control experiment showed that expression of the *cobU-lacZ^+^* fusion depended only on metabolically active cells. Second, we performed a similar experiment, but this time, we embedded strain JE27702 (*metE metH cobU-lacZ^+^*) in the agar plate. The absence of the Cba-dependent methionine synthase MetH enzyme in strain JE27702 made the strain unresponsive to Cbas and would only grow if methionine was present in the medium. As seen in Fig. 4, panel 2, the blue color was absent because strain JE27702 could not grow due to the absence of methionine synthase activity in the strain. In a third experiment, we spread strain JE27521 (*metE*^+^ Δ*cobS*) on the surface of a plate in which the Cba-auxotroph (JE27701) was embedded. In this experiment, we did not observe any blue color because the *lacZ*^+^ gene carried by strain JE27701 was not expressed since strain JE27701 spread on the surface of the agar did not make Cbas (Fig. 4, panel 3). This result strongly suggested that strain JE7087 was the source of Cba consumed by the Cba auxotroph.

Lastly, when we spread strain JE27521 (*metE^+^* Δ*cobS*) on the surface of an agar plate containing strain JE27702 (*metE* Δ*metH cobU-lacZ^+^*), the former formed white colonies because strain JE27702 did grow because methionine was not available (Fig. 4, panel 4). Panel 5 of Fig. 4 illustrates the results of the above experiments.

### Extracellular Cba levels support different Cba-dependent functions in *S*. Typhimurium

To quantify the level of excreted Cbas, we monitored the growth kinetics of cells under conditions that demanded different levels of Cbas, namely, methionine biosynthesis (requires low Cba levels) or ethanolamine catabolism (requires high Cba levels).

#### L-methionine biosynthesis

**Fig 5:**
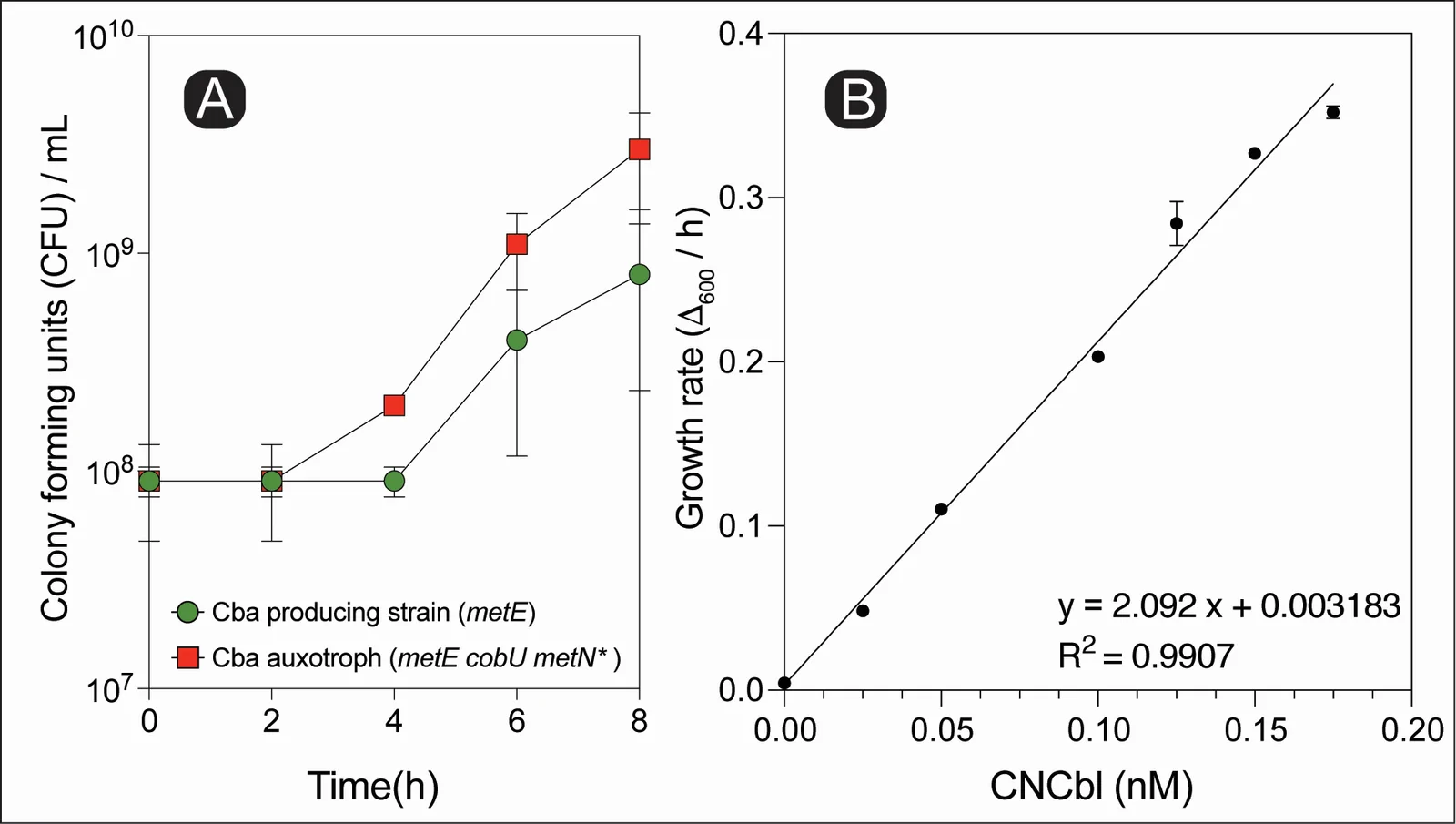
Cba excretion by the Cba-producing strain occurs early during growth. (**A**) Growth kinetics of strains JE7087 (Cba producer, green circles) and JE27701 (Cba auxotroph, red squares) grown in NCE minimal medium containing glycerol (22 mM), (CN)_2_Cbi (10 nM), and DMB (300 μM). The asterisk on the genotype of strain JE27701 indicates the presence of chromosomal null alleles of L-Met transporters Δ*yjeH* and Δ*leuE*. Complete genotypes are reported in Table 2. The experiment was done three times with technical duplicates. (**B**) Linearity of the growth response to the concentration of cyanocobalamin (CNCbl). Each point represents the variation of three technical replicates. This experiment was repeated three times with representative results shown. The strain used to generate the standard curve shown in panel **B** was JE8214 (*metE cobS*). Statistical analysis was performed using Prism (v10, GraphPad).

We determined the growth kinetics of a Cba producer and a Cba auxotroph under conditions that demanded Cba-dependent L-Met biosynthesis. For this purpose, we quantified viable cell counts at different time points of growth of a liquid co-culture in minimal medium containing glycerol (22 mM) as the sole source of carbon and energy plus Cba precursors (CN)_2_Cbi (10 nM) and DMB (300 μM). Surprisingly, we observed a longer lag phase for the culture of the Cba-producing strain (4 h) than that of the Cba auxotroph (2 h); however, after the onset of exponential growth, the growth rate was very similar for both strains (Fig. 5A). This result suggested that 2 h after inoculation, the Cba auxotroph acquired sufficient Cba to initiate exponential growth. We considered two possible sources of Cbas: (i) Cba excretion by the producing strain or (ii) presence of Cbas in the medium because Cba-producing cells lysed. We favored the first possibility because we suspect the number of cells lysing in fresh medium at the early phase of growth to be minimal. However, under the condition tested (L-Met biosynthesis), we did not rule out cell lysis because the requirement of *S*. Typhimurium for Cba for optimal MetH function is ~0.2 nM for *S*. Typhimurium (Fig. 5B).

#### Ethanolamine catabolism

*S*. Typhimurium has three known Cba-dependent enzymes: (i) ethanolamine ammonia-lyase (EAL) (25), (ii) propanediol dehydratases (26), and (iii) MetH (27). EAL activity allows *S*. Typhimurium to utilize ethanolamine as a carbon, nitrogen, and energy source (28, 29), but to catabolize ethanolamine, *S*. Typhimurium requires high levels of Cbas (Fig. 5B vs Fig. S3A). We increased the demand for Cba by growing the Cba auxotroph in medium supplemented with ethanolamine as the source of carbon and energy. In addition, the medium was supplemented with (CN)_2_Cbi and DMB to facilitate aerobic Cba biosynthesis by the Cba-producing strain. At least a >250-fold increase in the concentration of Cbl in the medium was required to optimally utilize ethanolamine as the carbon and energy source compared to the amount of Cbl needed for methionine biosynthesis (compare data in Fig. 5B; Fig. S1A).

**Fig 6:**
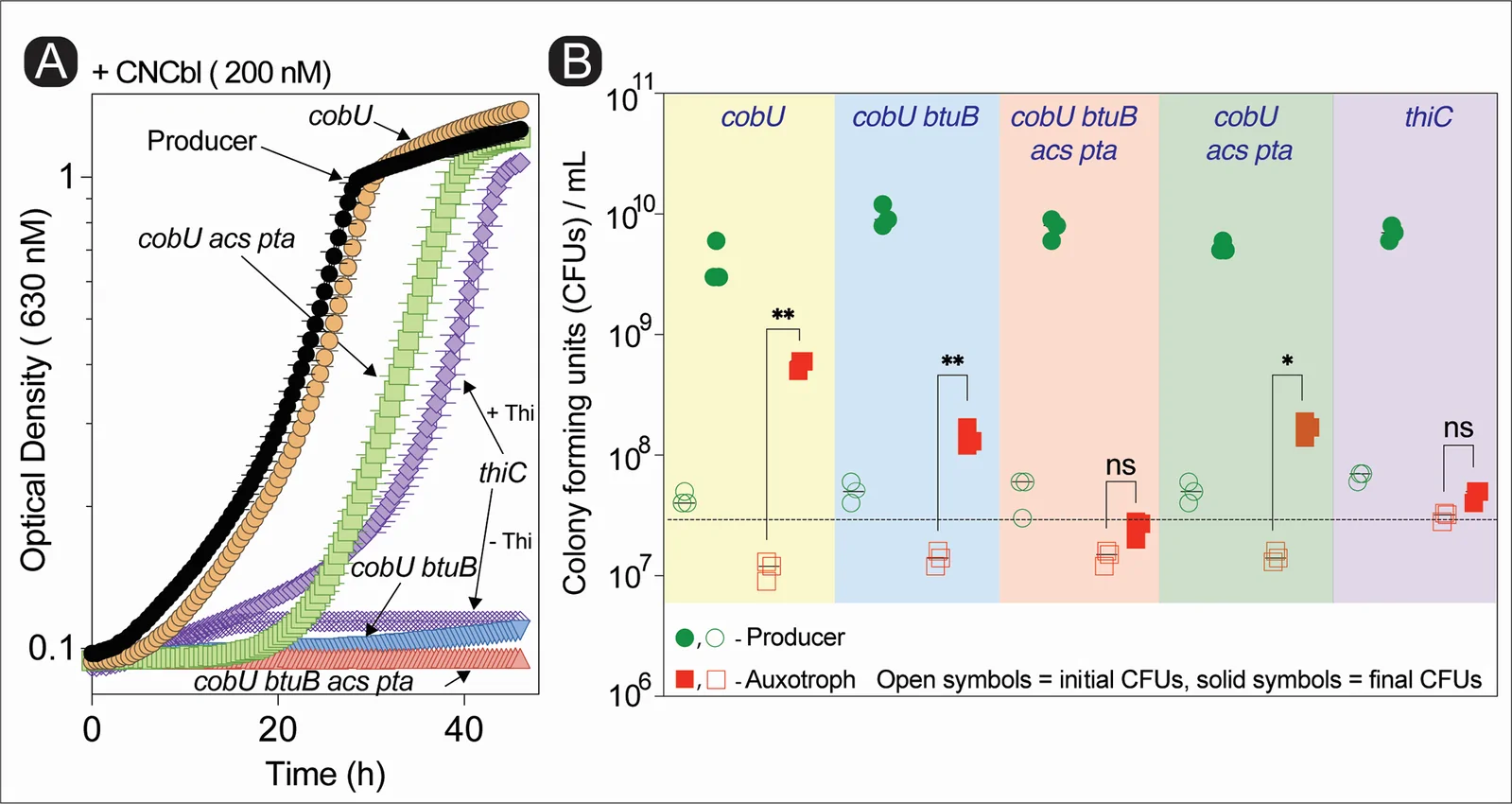
The extracellular concentration of Cbas in a co-culture is high enough to support ethanolamine catabolism. All cultures were grown in NCE minimal medium supplemented with ethanolamine (90 mM) and L-Met (300 μM). (**A**) Single-strain cultures of all strains used for co-cultures were grown in the presence of CNCbl (200 nM) in the medium. Growth of strain JE18413 required the medium to be supplemented with thiamine (200 nM). (**B**) Co-cultures were grown supplemented with (CN)_2_Cbi (200 nM) and DMB (300 μM), which needed to be converted to Cbas for cells to grow. Strain JE7087 (*araB metE*) was used as the Cba or thiamine-producing strain in all the co-cultures. The relevant genotype of the auxotrophic strain used in each co-culture is shown at the top. All auxotrophs carried additional *araB* and *metE* alleles in the chromosome. Initial cell counts are shown in open symbols, and cell counts during the stationary phase of growth (~48 h) are shown as filled symbols. Overnight cultures for the inoculum were grown in LB except for the *thiC* co-culture, for which overnight cultures were grown in NB to minimize thiamine carryover. Antibiotic markers were used to determine viable cell counts of each strain. A paired Student’s *t*-test was performed to determine statistical significance (ns, not significant; *P < 0.05, **P < 0.01). Each experiment was performed with biological and technical triplicates, with a representative result shown.

Figure 6 shows that a strain that converted (CN)_2_Cbi into a Cba excreted some of the latter into the medium when grown on ethanolamine as the source of carbon and energy. In Fig. 6A, we show the growth kinetics in response to CNCbl of monocultures of all the strains used in this study. To control for the possibility that cell lysis rather than excretion was the source of Cbas, we used a thiamine auxotroph as a control strain. Growth of the thiamine auxotroph would indicate the presence of thiamine in the medium and that Cbas were also released. All strains carried a chromosomal null allele of *metE* to force cells to use the Cba-dependent methionine synthase MetH to make methionine. To limit the use of Cba to ethanolamine catabolism, we provided cultures with methionine. We examined the ability of the strains to catabolize ethanolamine, since high levels of Cba were needed by the ethanolamine ammonia-lyase that initiates the breakdown of this amino alcohol. The strains used in the experiments were (i) JE7087 (*metE*, i.e., the Cba producer); (ii) JE1736 (*metE cobU*, i.e., a Cba auxotroph); (iii) JE28339 (*metE cobU btuB*, i.e., a Cba auxotroph unable to take up corrinoids); (iv) JE28428 (*metE cobU btuB acs pta*, i.e., a Cba auxotroph that cannot take up corrinoids nor can it activate acetate); (v) JE28303 (*metE cobU acs pta*, i.e., Cba auxotroph that cannot grow on acetate); and (vi) JE18413 (*metE thiC*, i.e., a thiamine auxotroph control).

##### Growth behavior of single cultures

The results shown in Fig. 6A show that to grow on ethanolamine as the sole source of carbon and energy, *S*. Typhimurium needs to take up a Cba (in this case CNCbl, inverted blue triangles vs black and orange circles). The behavior of strain JE28303 was consistent with the reported need for acetyl-CoA synthetase (Acs) and phosphotransacetylase (Pta) activities during growth on ethanolamine (green squares) (30). The thiamine auxotroph behaved as expected (open vs closed purple diamonds)

##### Growth of strains in co-cultures

In Fig. 6B, we show results obtained with co-cultures of the Cba producing strain (JE7807) with each one of the strains used in Fig. 6A. In these experiments, we quantified CFUs for each member of the co-cultures at the time of inoculation (open symbols) and after ~48 h of growth at 37°C (solid symbols). The medium used in these experiments was the same we used for the single-strain culture experiment (Fig. 6A), except CNCbl was replaced with precursors (CN)_2_Cbi and DMB. Across all panels, the Cba producing strain reached the highest CFUs/mL of all co-cultures after 48 h of incubation. However, important differences were observed among derivatives of the Cba auxotroph. The yellow panel shows an almost two-order-of-magnitude increase in the CFUs/mL of the Cba auxotroph after a 48-h incubation period. To test whether such growth was due to the presence of Cbas in the medium, we repeated the experiment using strain JE28339, which could not take up corrinoids due to the absence of the BtuB protein (*metE cobU btuB*) responsible for corrinoid transport across the outer membrane (Fig. 6B, blue panel). The absence of BtuB had a profound negative effect on growth, since the number of viable cells of strain JE28339 after 48 h of incubation was only one-order-of-magnitude higher than that at the time of inoculation (Fig. 6B, blue panel). The observed growth of strain JE28339 was unexpected since the strain lacked the BtuB transporter. We suspected that an additional source of carbon and energy was present in the co-culture, and that such source was likely excreted by the Cba producer. In previous work, our laboratory reported that during growth of *S*. Typhimurium on ethanolamine, cells excrete acetate in the millimolar range (30). To investigate whether the residual growth of the strain JE28339 (red squares, blue panel) was due to acetate excreted by the Cba producer, we used a Cba auxotroph that could not catabolize acetate due to the absence of the Acs and the housekeeping Pta enzymes, in addition to *cobU* and *btuB* null alleles (JE28428, *metE cobU btuB acs pta*; Fig. 6B, red panel). This strain failed to grow on ethanolamine when co-cultured with a Cba producer because the Cba or acetate in the medium could not be utilized by the Cba auxotroph (Fig. 6B, salmon panel). This reduction in CFUs supported the idea that growth of the JE28339 strain was due to the presence of acetate in the medium. About one order of magnitude increase in CFU per milliliter of strain JE28303 (*metE cobU acs pta*; Fig. 6B, green panel) in co-culture verified the Cba-dependent growth of a Cba auxotroph in co-culture.

##### Cbas in the medium are excreted, not released by cell lysis

To test whether Cbas were present in the medium because cells of the Cba-producing strain lysed, we looked for thiamine in the co-culture. We posited that if cells lysed, they would release growth factors such as thiamine into the medium. We tested for the presence of thiamine in the medium using a thiamine auxotroph (JE18413) that carried a null allele of 4-amino-5-hydroxymethyl-2-methylpyrimidine phosphate synthase gene (*thiC*, a gift from D. Downs) (31). We observed that the requirement of thiamine in a minimal medium containing ethanolamine as the source of carbon and energy was comparable to that of the Cba requirement by a Cba auxotrophic strain (Fig. S2A and B). However, when a *thiC* strain was co-cultured with the Cba- and thiamine-producing strain (JE7087), the *thiC* strain failed to grow after ~48 h of incubation (Fig. 6B, purple panel). This result suggested that, if thiamine pyrophosphate or its precursors were present in the medium, they were at concentrations below those required for growth of strain JE18413, making it unlikely that Cbas were the result of cell lysis.

### Minimal cobamide concentration required for growth

To understand the relative minimum requirement of Cba for growth with ethanolamine as the source of carbon and energy, we grew strain JE1736 (*metE cobU*) at varying concentrations of CNCbl. We observed that >0.5 nM of CNCbl was required to obtain visible growth (OD_630_ <0.2) on ethanolamine, and ~50 nM was needed to achieve an OD_630_of ~1.2 (Fig. S3A). In contrast, when growth required Cba-dependent methionine synthase MetH function, we observed growth at CNCbl concentrations as low as ~25 pM (Fig. 5B), with >150 pM CNCbl needed to reach a growth rate >0.3 DOD_600_ h^−1^ (Fig. 5B).

### How much cobamide is excreted?

To evaluate the extracellular concentration of Cbas that the Cba producer strain JE7087 excreted, individual cultures of the Cba producer (JE7087) and co-cultures of it with a Cba auxotroph (JE27701 or JE28303) were grown on ethanolamine or glycerol as the source of carbon and energy (Table 1).

**TABLE 1:** Extracellular Cba concentrations in cultures grown under different conditions

Strains in the culture	Growth stage	Carbon source	(CN)_2_Cbi (nM)	Extracellular Cba (pM (± SD)
Cba producer alone	Log phase	Glycerol	10	5.72 (±0.1)
Cba producer alone	Early stationary	Glycerol	10	7.34 (±0.2)
Cba producer alone	Late stationary	Glycerol	10	3.33 (±0.1)
Co-culture of Cba producer plus Cba auxotroph	Late stationary	Glycerol	10	2.36 (±0.1)
Cba producer alone	Early stationary	Glycerol	75	31.81 (±1)
Cba producer alone	Log phase	Ethanolamine	200	30.0 (±3)
Culture of producer	Early stationary	Ethanolamine	200	39 (±2)
Culture of producer	Late stationary	Ethanolamine	200	22 (±3)
Co-culture producer/auxotroph	Late stationary	Ethanolamine	200	25 (±3)

Cultures were harvested at different stages of growth, and extracellular corrinoids were partially purified from spent cultures using a SepPak C18 cartridge as described in Materials and Methods. To measure the relative extracellular Cba concentration in each culture, we used a standard of the change in growth as a function of the concentration of CNCbl (Fig. 5B). To ensure that growth was due to extracted Cbas, we used the Cba auxotrophic strain JE8214 (*metE cobS*) in our bioassay. We observed a relatively low concentration of extracellular Cbas (<10 pM for glycerol-grown cultures and <40 pM for ethanolamine-grown cultures) when the Cba precursor (CN)_2_Cbi was provided (Table 1). However, we observed a gradual increase in extracellular Cba from the logarithmic growth phase to the early stationary growth phase, which then decreased during the stationary growth phase. To test whether the apparent increase of extracellular Cbas in ethanolamine cultures was due to the higher concentration of (CN)_2_Cbi provided (200 nM), we increased the (CN)_2_Cbi concentration from 10 to 75 pM in minimal glycerol medium. This change in (CN)_2_Cbi concentration increased the extracellular Cba concentration from ~7.3 to 31.8 pM. The extracellular Cba concentration in early-stationary phase co-cultures when grown in the presence of a Cba auxotroph (JE27701 and JE28303 for cultures grown on glycerol and ethanolamine, respectively) was comparable to pure cultures of the Cba producer at a similar growth stage (3.3 pM vs 2.36 pM for glycerol cultures vs 22 pM vs 25 pM for ethanolamine cultures, respectively).

### *S*. Typhimurium excretes different cobamides

**Fig 7:**
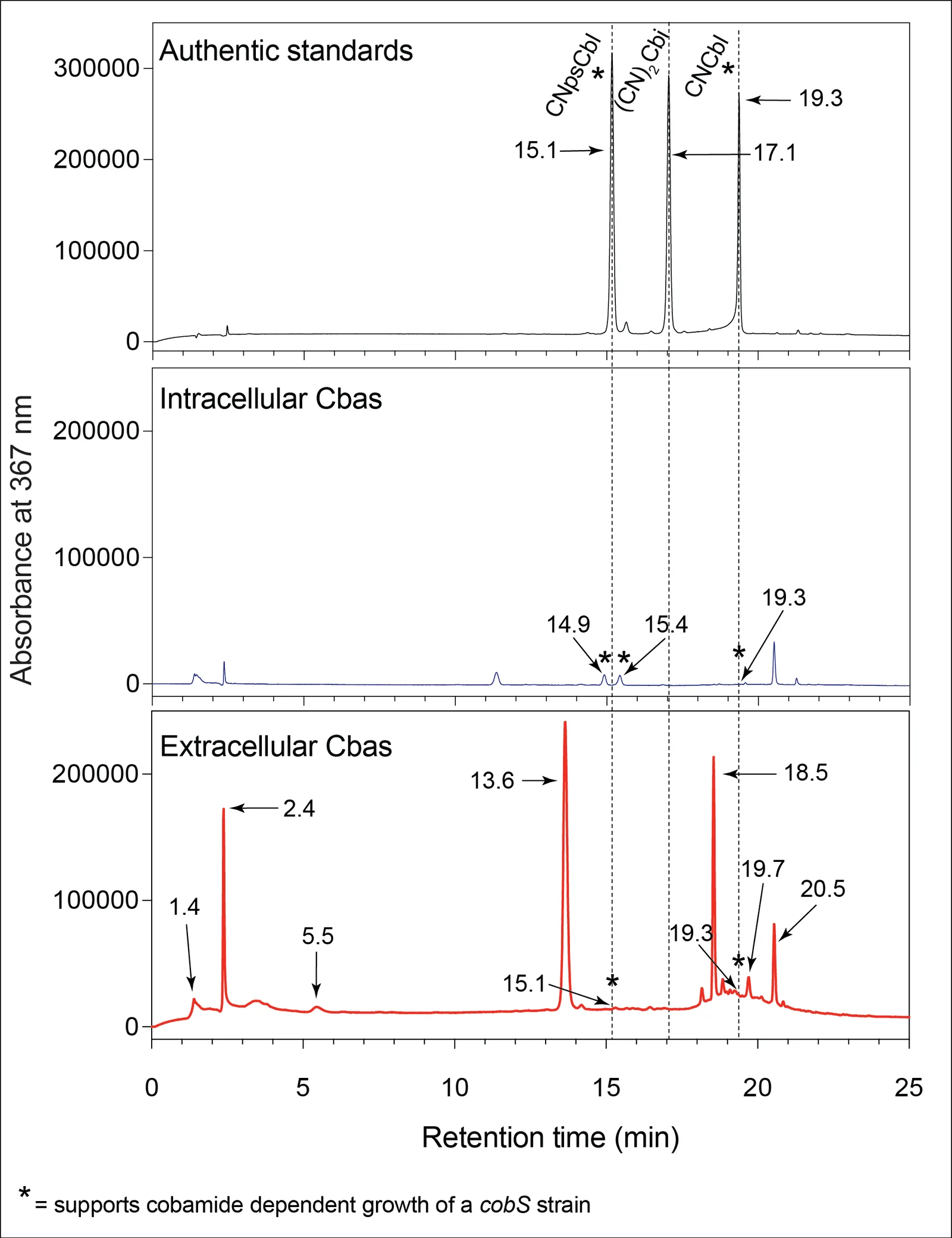
Different Cbas were detected in the culture medium. *S*. Typhimurium strain JE10079 (Δ*araB*) strain was grown in minimal medium (250 mL) supplemented with ribose (50 mM), 1,2-propanediol (10 mM), and CoCl_2_ (4 μM) under anaerobic conditions. The culture was harvested at the early stationary phase of growth, and extracellular and intracellular corrinoids were extracted as described in Materials and Methods. Cbas were separated using RP-HPLC. The experiment was repeated three times with a representative chromatogram shown. Retention time of standards shown as dotted lines: CNpsCbl, cyanopseudocobalamin, (CN)_2_Cbi, dicyanocobinamide, and CNCbl, cyanocobalamin. Cbas were detected using a bioassay as described in Materials and Methods and Fig. S4.

We used reverse-phase high-performance liquid chromatography (RP-HPLC) to identify excreted Cbas. For this purpose, we grew *S*. Typhimurium anaerobically in a minimal medium supplemented with ribose as the main source of carbon plus cobalt chloride (CoCl_2_) to ensure that cobalt levels did not limit Cbas biosynthesis. After extracting corrinoids from spent medium using a SepPak C18 Plus cartridge, samples were analyzed by RP-HPLC on a C18 column as described in Materials and Methods. To identify Cbas, each peak was collected and analyzed with the bioassay that used the Cba auxotrophic strain JE8214 (*metE cobS*). Compounds that eluted at ~1.4, 2.4, 5.5, 13.6, 18.5, 19.7, and 20.5 min post-injection did not support growth of the bioassay strain JE8214 (Fig. 7; Fig. S4). We used two different Cba standards that *S*. Typhimurium is known to synthesize. Cbl, which eluted at 19.3 min (Fig. 7, top panel), and psCbl, which eluted ~15.1 min post-injection (Fig. 7, top panel). We detected the presence of two peaks in the intracellular corrinoid pool of *S*. Typhimurium eluting at 14.9 and 15.4 min, and both compounds supported growth of the bioassay strain (JE8214, Fig. S4). We surmised that these two peaks were likely two different cyanation states of psCbl. The intracellular corrinoid pool contained one additional complete Cba that eluted 19.3 min post-injection, which supported growth of strain JE8214 (Fig. 7; Fig. S4) even though insufficient amounts of the Cba were present in the sample; hence, it was not detected by the instrument. The chromatogram of excreted Cbas showed activity at 15.1 and 19.3 min post-injection, which aligned with retention times of psCbl and Cbl, respectively.

### Cba excretion allows *S*. Typhimurium to form Cba-dependent colonies aerobically

**Fig 8:**
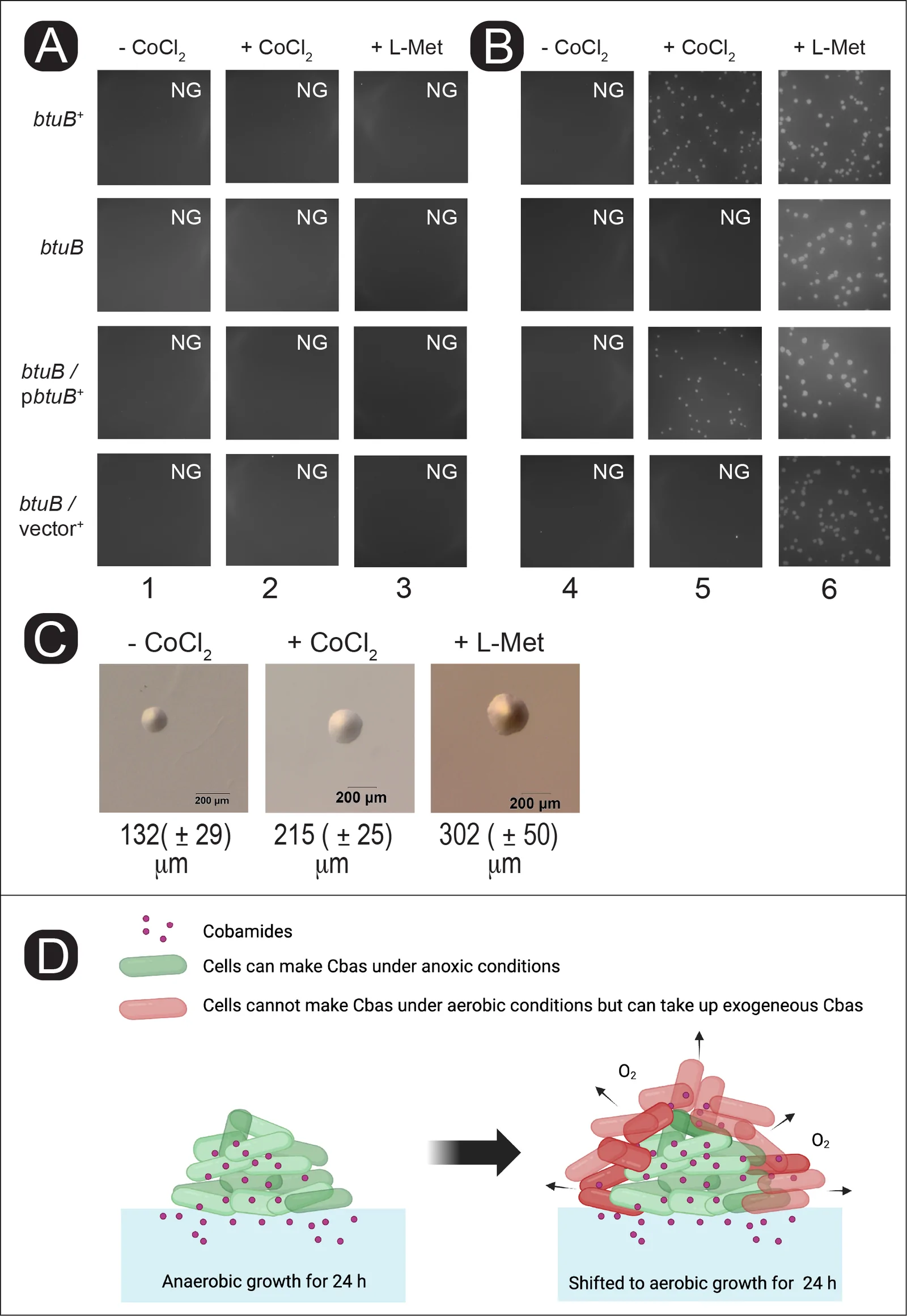
*De novo* Cba synthesis and excretion supported Cba-dependent growth of colonies under aerobic conditions. Cells from strains JE6583 (*araB metE*), JE1299 (*araB metE btuB*), JE1626 (*araB metE btuB*/pBtuB^+^), and JE28642 (*araB metE btuB*/vector) were spread on NCE agar plates supplemented with sorbitol (30 mM) and 1,2-propanediol (10 mM). L-Met (300 μM) or CoCl_2_ (4 μM) was added to the medium as indicated. Plates were incubated for 24 h under anoxic conditions at 37°C (**A**) and incubated under aerobic conditions for an additional 24 h (**B**) after shifting from anaerobic to aerobic conditions. NG, no visible growth to the naked eye. Each experiment was replicated three times, with a representative result shown. (**C**) Cells from strain JE6583 (*metE*) were incubated for 24 h under anoxic conditions, and colonies were observed under a dissecting microscope with a 45× total magnification. The size of 10 colonies was measured for each biological replicate, with a representative colony shown from each condition. Each experiment was repeated three times. (**D**) Working model for aerobic Cba-dependent colony formation; a microcolony was formed during anaerobic growth of cells. Upon shifting to aerobic conditions, Cbas were synthesized *de novo* within an “anoxic core” of the microcolony and excreted, which allowed cells in the periphery of the anoxic core to grow even when exposed to oxygen. The illustration in panel D was created with BioRender.com.

*S*. Typhimurium synthesizes Cbas *de novo* only under anaerobic conditions (10). We questioned whether Cba excretion by *S*. Typhimurium would support colony formation under aerobic conditions if growth depended on Cbas. We tested this idea on a 1.6% (wt/vol) agar surface demanding that strain JE6583 (*metE*, Cba producer) initiate colony formation under anaerobic conditions. We determined that a 16-h incubation period under anaerobic conditions did not support colony formation (Fig. S5A), even after an additional 32 h of incubation under aerobic conditions, unless L-Met was added to the medium (Fig. S5A). These results suggested that 16 h of incubation under anaerobic conditions was not enough for a microcolony to excrete enough Cbas to grow under aerobic conditions. In contrast, a 24-h incubation period under anaerobic conditions allowed strain JE6583 (Cba producer) to form microcolonies that produced Cbas after shifting to aerobic conditions (Fig. 8).

Experiments performed after a 24-h incubation period under anaerobic conditions were done using agar plates supplemented with CoCl_2_ to ensure *de novo* Cba biosynthesis. After a 24-h incubation period, we did not observe any colonies on the agar surface (Fig. 8A, *btuB*^+^, column 2), but after an additional 24-h incubation period under aerobic conditions, colonies appeared (Fig. 8B, *btuB*^+^, column 5). Since the medium was devoid of corrinoids or methionine, we assumed that the observed aerobic colony formation represented Cba-dependent growth, and cells that were initially grown under anaerobic conditions were responsible for *de novo* synthesis and excretion of Cbas, which supported cell growth in the presence of oxygen. In other words, the increase in colony size was due to the continuous production of Cbas by what we call the “anoxic core” of the colony.

To test whether cells divided because they took up Cbas excreted by the anoxic core, we substituted strain JE1299 (*metE btuB*, a Cba uptake-deficient strain) for strain JE6583 (*metE btuB^+^*). Strain JE1299 failed to form visible colonies under aerobic condition (Fig. 8B, *btuB* column 5), consistent with the idea that aerobic growth of strain JE6383 depended on extracellular Cbas. Growth of the *btuB* strain was restored when the strain carried a plasmid encoding the wild-type allele of *btuB* (Fig. 8B, column 5). These *in vivo* results confirmed that Cbas were excreted and retaken up from the medium. To verify that Cbas in the medium were synthesized by anaerobically growing cells, we repeated the experiment on a medium devoid of cobalt. The presence of cobalt was required for aerobic colony formation (Fig. 8B, column 4 vs column 5), further supporting the conclusion that excreted Cbas were synthesized *de novo*.

We also measured the average size of the colonies after 24 h of incubation in a medium supplemented with CoCl_2_ (215 ± 25 mm). The average size of these colonies was approximately 70% the size of a colony growing on the medium supplemented with methionine (302 ± 50 mm) (Fig. 8C). We observed some microscopic residual growth of colonies (132 ± 29 mm) in a medium where CoCl_2_ was not added (Fig. 8C). This was likely due to trace amounts of cobalt ions in the medium. The intent of Fig. 8D is to illustrate how we think Cba production by anaerobically growing cells drives continuous colony growth after shifting to aerobic conditions.

### Genetic evidence supports Cba biosynthesis in an anaerobically growing colony

**Fig 9:**
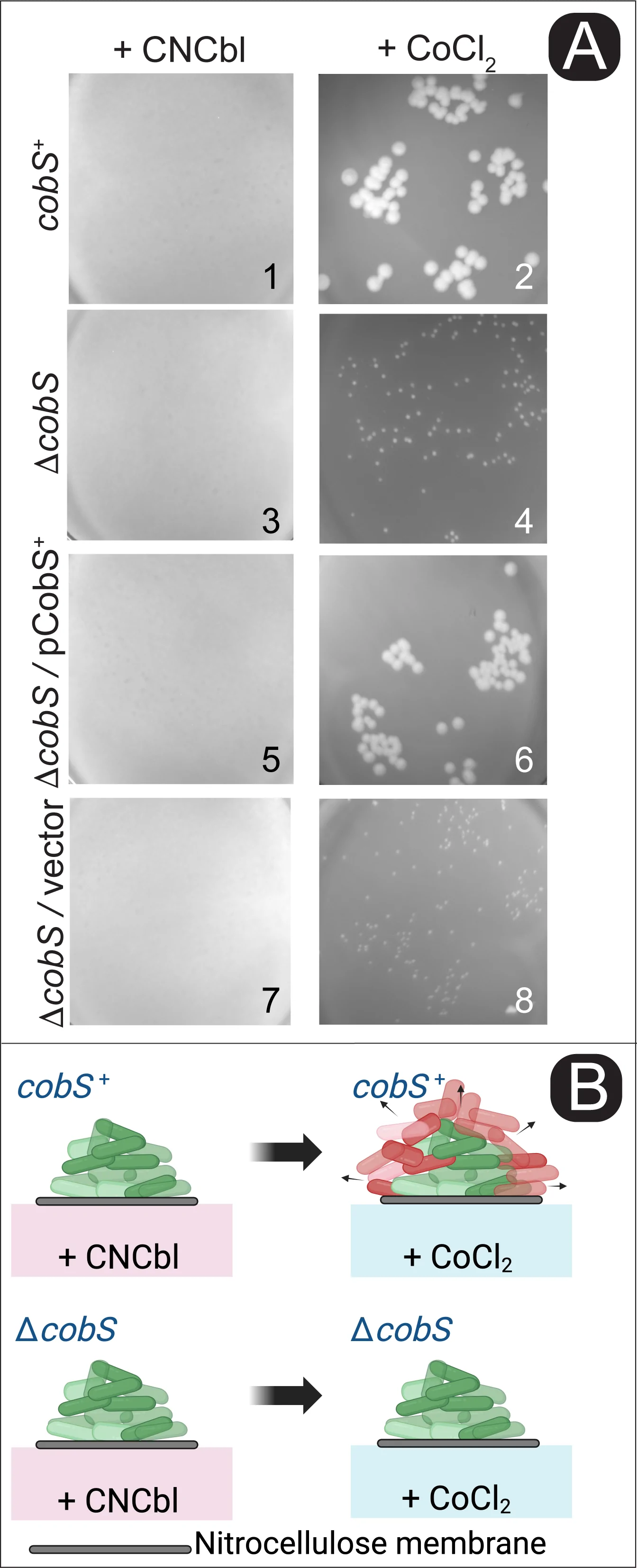
In *S*. Typhimurium *de novo* Cba biosynthesis is essential for robust colony growth. (**A**) Cells from strains JE6583 (*cobS^+^*), JE8248 (*cobS*), JE15888 (*cobS*/pCobS*^+^*), and JE16852 (*cobS*/vector) were grown on a nitrocellulose membrane kept on the surface of an NCE agar plate containing sorbitol (30 mM), 1,2-propanediol (10 mM), L-(+)-arabinose (250 μM), and CNCbl (50 pM). Plates were incubated for ~24 h at 37°C, and membranes were transferred onto a similar agar medium without CNCbl but with CoCl_2_ (4 μM). Plates were further incubated for 4 days at 37°C. Each experiment was replicated three times, with a representative result shown. (**B**) Working model for aerobic Cba-dependent colony formation; a microcolony was formed by incubating cells on a nitrocellulose membrane placed on a medium containing CNCbl. A strain capable of *de novo* biosynthesis of Cba (e.g., *cobS^+^*) showed maximal aerobic growth due to biosynthesis and excretion of Cbas. The illustration shown in panel B was created with BioRender.com.

We used a genetic approach to lend support to the idea that *de novo* biosynthesis of Cbas in an anaerobically growing colony supports growth of the colony after shifting to aerobic conditions. For this purpose, we grew strain JE6583 (*metE*, Cba producer) on a nitrocellulose membrane resting on minimal medium containing sorbitol as the main source of carbon and energy plus 1,2-propanediol. The purpose of 1,2-propanediol was to induce the Cba biosynthetic genes (32). We added a low concentration of CNCbl (50 pM) to the medium to satisfy the Cba requirement for the synthesis of methionine. The low concentration of CNCbl used in the experiment would allow survival but initiate colony formation, but not to the extent of yielding visible colonies. After 24 h of incubation under aerobic conditions at 37°C, we did not observe any colonies (Fig. 9A, panel 1). The nitrocellulose membrane was transferred to a medium devoid of Cbas but supplemented with enough CoCl_2_ (4 μM) for cells to synthesize Cbas *de novo* if a sufficiently large microcolony had arisen. Notably, after an additional 4 days of incubation at 37°C under aerobic conditions, colonies appeared on the membrane (Fig. 9A, panel 2).

These results suggested that anaerobically grown microcolonies arose during the initial 24-h incubation period when cells used the limited amount of CNCbl in the medium and shifted to *de novo* Cba biosynthesis when provided with sufficient cobalt ions. To demonstrate that *de novo* synthesized Cbas supported the robust growth observed after the 4-day incubation period (Fig. 9A, panel 2), we blocked the penultimate step of Cba biosynthesis (catalyzed by CobS, Fig. 1B) in strain JE6583 (yielding strain JE8248) and predicted that growth of strain JE8248 would be greatly reduced. After incubating strain JE8248 on a medium containing CNCbl (Fig. 9A, panel 3) for 24 h under aerobic conditions, the membrane was transferred onto the medium containing CoCl_2_. As expected, visible growth of JE8248 was notably impaired compared to that of strain JE6583 (*metE cobS^+^*) (Fig. 9A, panel 4), indicating that *de novo* Cba biosynthesis happened inside a colony growing aerobically. Panels 5–8 of Fig. 9 were control experiments where we showed that the lack of growth observed in panel 4 can be corrected by a plasmid carrying the wild-type allele of *cobS* but cannot be complemented by the empty vector (panel 8). Figure 9B summarizes schematically the data presented in Fig. 9A.

### Anaerobically grown cells can keep up with increased demands for *de novo* Cba biosynthesis

To test whether anaerobically grown cells could satisfy the need for higher levels of Cbas required to support Cba-dependent metabolism, we grew *S*. Typhimurium with ethanolamine as the source of carbon and energy. Ethanolamine catabolism is initiated by the Cba-dependent ethanolamine ammonia-lyase (EC 4.3.1.7) encoded by the *eutBC* genes of the ethanolamine utilization (*eut*) operon (33). However, to generate enough carbon and energy, the cell needs high levels of Cbas. We posited that if anaerobically growing cells could support colony formation on plates supplemented with ethanolamine as the sole source of carbon and energy, it would imply that the extracellular level of Cbas was high.

**Fig 10:**
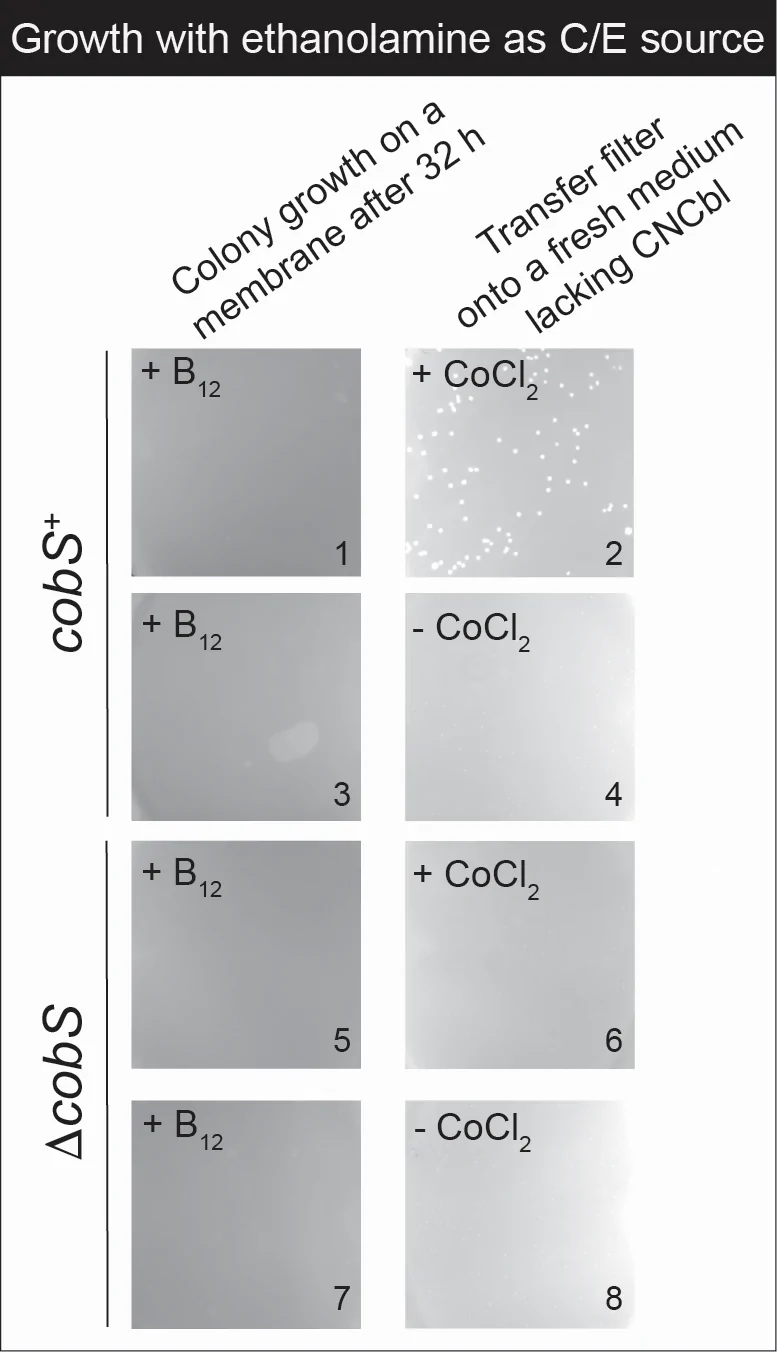
*De novo* Cba synthesis supported Cba-dependent visible growth of colonies in ethanolamine. Cells from strains JE6583 (*cobS^+^*) or JE8214 (*cobS*) were grown on a nitrocellulose membrane kept on the surface of an NCE agar plate containing ethanolamine (90 mM), methionine (300 μM), sodium tetrathionate (40 mM), and CNCbl (10 nM); plates were incubated for ~32 h at 37°C. Membranes were transferred onto a new agar medium without CNCbl but with or without CoCl_2_ (4 μM) as indicated, and incubated further for 4 days at 37°C. Each experiment was replicated three times, with a representative result shown.

We allowed strain JE6583 (*metE eut^+^*) to grow for 32 h under aerobic conditions on a nitrocellulose membrane placed on top of an agar plate containing minimal medium supplemented with ethanolamine (90 mM) and CNCbl (10 nM) (Fig. 10, left column). We note that the low concentration of CNCbl used could only support slow cell growth. Unsurprisingly, no colonies were visible after 32 h of incubation (Fig. 10, panel 1 and 3). At this point, the membrane was transferred onto minimal medium plates that lacked Cbas but either contained CoCl_2_ or not (Fig. 10, panels 2 and 4); after the membrane transfer, all plates were incubated under aerobic conditions. Since ethanolamine is a non-fermentable carbon source, we included tetrathionate as an electron acceptor, which has been shown to allow *S*. Typhimurium to respire ethanolamine under anaerobic conditions (34). We reasoned that if *de novo* Cba biosynthesis stopped after a shift to aerobic conditions, but colonies continued to grow, it would imply that cells accumulated enough CNCbl during the anaerobic stage of the experiment. If this were the case, colony formation would happen in the presence of air regardless of the presence or absence of CoCl_2_ in the medium; that, however, was not the case. In fact, CoCl_2_ was required for continuous cell growth after the transfer of the membrane to medium devoid of CNCbl (Fig. 10, panels 1 through 4). Furthermore, a functional Cba biosynthetic pathway was needed for colony formation, as no visible colonies were observed in strain JE8214 (*metE cobS*, a Cba auxotroph) (Fig. 10, panels 5 through 8). Collectively, our results support the idea that *S*. Typhimurium excreted *de novo* synthesized Cbas by an as-yet-to-be-identified mechanism.

### B_12_ utilization functions are not needed for Cba excretion across the inner membrane

**Fig 11:**
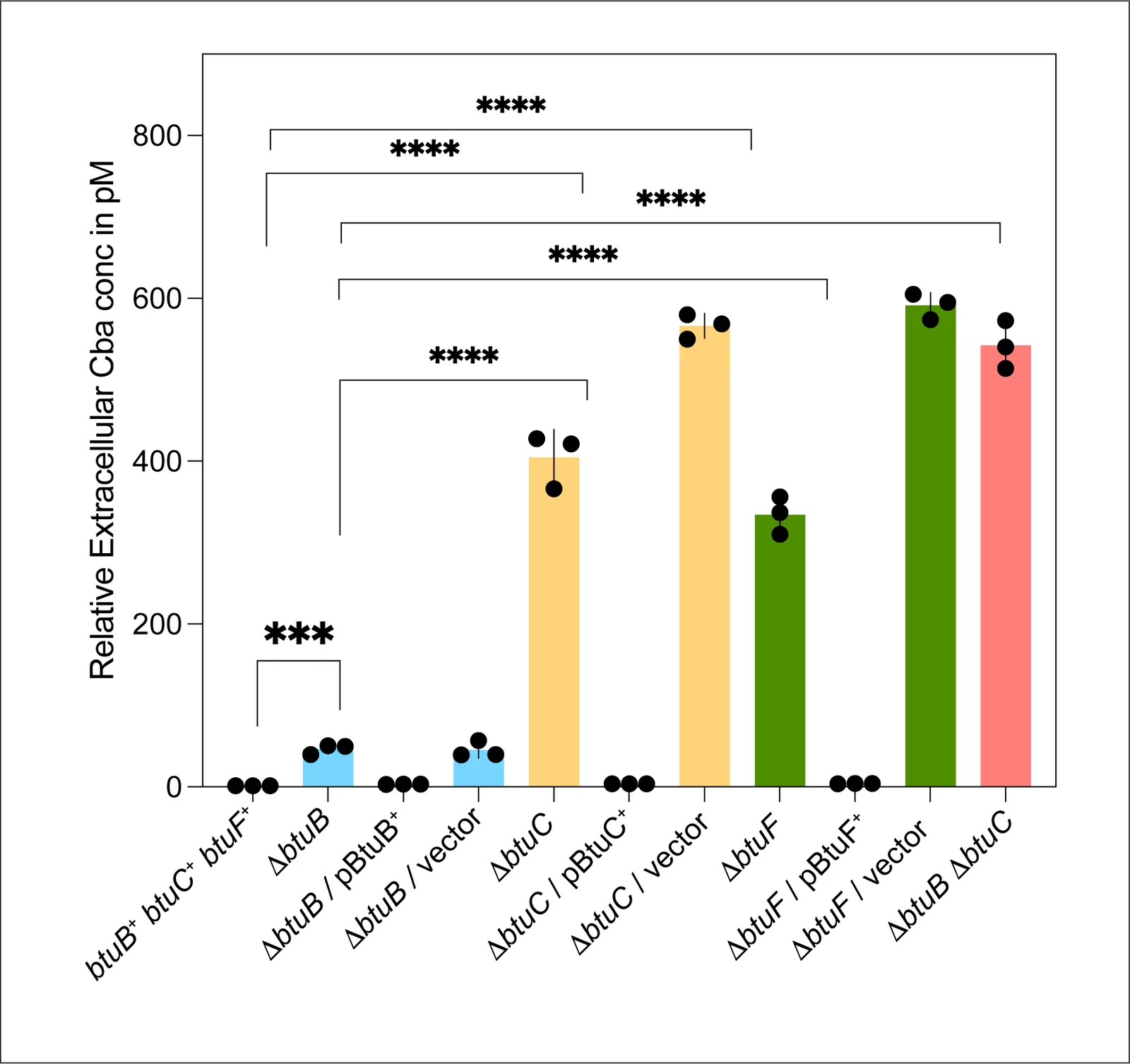
*S*. Typhimurium strains devoid of corrinoid transporters maintained high extracellular levels of Cbas. Relative extracellular concentrations of cultures grown under anoxic conditions were calculated. The strains used in these experiments were JE6583 (*btuB^+^ btuC^+^ btuF^+^*), JE1299 (Δ*btuB*), JE1626 (Δ*btuB*/pBtuB^+^), JE28642 (Δ*btuB*/vector), JE16001 (Δ*btuC*), JE28638 (Δ*btuC*/pBtuC^+^), JE28639 (Δ*btuC*/vector), JE28571 (Δ*btuF*), JE28607 (Δ*btuF*/pBtuF^+^), JE28640 (Δ*btuF*/vector), and JE28827 (Δ*btuB* Δ*btuC*). All the strains carried additional *araB* and *metE* alleles in the background. All cultures were grown in NCE minimal medium (10 mL) supplemented with ribose (50 mM), 1,2-propanediol (10 mM), and CoCl_2_ (4 μM) under anoxic conditions. 1,2-Propanediol was added to activate the expression of the Cba biosynthetic genes (+). Cultures were harvested at the early stationary phase of growth, and extracellular corrinoids were collected as described under Materials and Methods. Extracted Cbas were diluted as needed, and a Δ*cobS* (JE8214, *araB metE* Δ*cobS*) strain was grown on extracted Cbas. The growth rate of the Δc*obS* strain upon exposure to different Cba extracts was measured, and relative extracellular Cba concentration was calculated using the standard curve shown in Fig. 5B. Statistical significance was determined using unpaired *t*-test with Prism (v10) software (GraphPad). ***P > 0.0002, ****P > 0.0001. Each experiment was repeated three times with three technical replicates, and a representative result is shown.

We quantified the extracellular concentration of Cba under *de novo* biosynthesis as a function of B_12_ utilization (Btu) functions. We did this to determine whether Btu functions affected in any way the concentration of extracellular Cbas. All strains used in these studies were grown under anaerobic conditions to allow *de novo* Cba synthesis to occur. The extracellular Cba concentration in a culture of a strain with all Btu transporters functional (i.e., *btuBCDF^+^*) was low (≤10 pM) (Fig. 11). Surprisingly, the extracellular Cba concentration increased ~30-fold in a strain carrying a null allele of the *btuB* gene (Fig. 11, blue bars), which encodes the outer-membrane Cba transporter. This increase in the extracellular Cba concentration was magnified when either the BtuF (periplasmic Cba carrier) or BtuC (inner membrane Cba transporter) protein was absent (>300-fold increase relative to the *btuBCDF^+^* strain), but the *btuB^+^* gene was present (Fig. 11, yellow and green bars, respectively). In all cases, ectopic expression of the wild-type allele of the *btu* gene tested lowered the Cba concentration to that of the *btuBCDF^+^* strain (+). To test whether the lower levels of extracellular Cba observed in the *btuB* strain compared to *the btuC* or *btuF* strains were due to export of Cbas via BtuB, we measured extracellular Cbas in a *btuB btuC* strain. Strain JE28827, which lacked both BtuB and BtuC transporters, was still able to excrete a significantly higher level of Cbas compared to the *btuB* strain (Fig. 11, salmon color vs blue color). To explain these observations, we hypothesized that excreted Cbas were taken up back into the cell by the Btu transport system and that the absence of each transporter somehow disrupted Cba reuptake, resulting in an increased extracellular concentration of Cba.

To test this idea, we monitored the Cba-dependent growth under anaerobic conditions when CNCbl was supplemented at different concentrations to force cell growth to depend on the uptake of Cba. To eliminate any contributions of *de novo* Cba biosynthesis, we deleted the majority of Cba biosynthetic genes (*cbiA-cobS*) in these strains (Fig. S6). Surprisingly, we observed that cells could take up CNCbl even at 250 pM in the absence of either BtuC or BtuF. However, in the absence of BtuB (the outer-membrane Cba transporter), the medium needed to contain CNCbl at >500 nM (Fig. S6). This indicated a >2,000-fold difference in CNCbl uptake efficiency in the absence of BtuC or BtuF vs BtuB. From these results, we concluded that excretion of Cbas did not require Btu functions, but uptake did.

## DISCUSSION

To our knowledge, this is the first report of Cba excretion by *S*. Typhimurium. This is an important finding because it advances our understanding of Cba metabolism in this human pathogen and because it suggests that Cba production by this bacterium may play an important role in its transition from an anaerobic to an aerobic environment depleted of Cbas. The idea of Cbas being exchanged in microbial communities is an attractive one that should be explored using microbes that are likely to interact with *S*. Typhimurium in the gut or in its free lifestyle environments. However, nutrient exchange is a more complex scenario, given that in such a situation, signals are needed to trigger the exchange. At this point, we need to better understand Cba excretion *per se*, and *S*. Typhimurium is a good model organism for this purpose since Cba synthesis is well understood in this human enteropathogen.

### *S*. Typhimurium excretes Cbas

The data supporting this conclusion are clear; however, we do not understand how Cbas are excreted. The question of cell lysis vs excretion is answered by the data presented in Fig. 5A, when we grew a Cba auxotroph with a Cba producer in co-culture and we noticed that logarithmic growth of the Cba auxotroph was initiated at a very early stage (~2 to 3 h), even earlier than the onset of logarithmic growth of the Cba producer. Importantly, during that period, the CFU per milliliter of either strain did not change, indicating that cell lysis was not occurring at any detectable level. Furthermore, the absence of thiamine (a nutrient needed at very low levels) in the extracellular milieu also argues against cell lysis (Fig. 6). Third, if cell lysis were the source of extracellular Cbas, we would expect the concentration of extracellular Cba to increase as the culture aged. In fact, the concentration of extracellular Cbas at different stages of culture growth slightly decreased as the culture aged (Table 1). Reuptake of excreted Cbas during the stationary phase of growth likely caused the apparent decrease of Cbas in the extracellular milieu. Collectively, our results support the idea of Cba excretion.

### In *S*. Typhimurium, Cba excretion requires anaerobically grown cells and cobalt ions

*De novo* Cba biosynthesis is inhibited by oxygen in *S*. Typhimurium, but prior to this work, it was not clear how long cells needed to grow under anaerobic conditions for them to support robust colony formation in the presence of air. The data we obtained show that *S*. Typhimurium needs at least 24 h of incubation under anaerobic conditions with cobalt ions in the medium. After 24 h of anaerobic growth, addition of Cbas to the medium was not needed because anaerobically grown cells produced and excreted Cbas to meet the needs of cells at the surface of the colony, which were growing in the presence of air (Fig. 8). That Cbas were synthesized *de novo* was confirmed by results obtained with a strain that lacked CobS, an enzyme involved in the late steps of Cba assembly (Fig. 9). Further support for the idea that excreted Cbas were required for growth of the testing strain was provided by the need for the outer-membrane BtuB corrinoid transporter. In the absence of BtuB, once we shifted cultures from anaerobic to aerobic growth conditions, colonies of the Cba auxotroph did not form (Fig. 8).

### Implications of our findings

We do not know why we must incubate *S*. Typhimurium for at least 24 h under anaerobic conditions before cells can grow in the presence of air and absence of exogenous Cbas. One explanation could be that the rate of O_2_ diffusion into the microcolony must be sufficiently reduced to allow activation of the Cba biosynthetic genes (32, 35–37). Our working model suggests that once a colony of *S*. Typhimurium reaches a threshold size, the microcolony constitutes what we call the “anoxic core,” which synthesizes Cbas *de novo*, some of which are excreted. Cba excretion by the anoxic core can support growth of cells on the outer layer of the colony, which in turn will become part of the anoxic core as cells divide and the colony grows even in the presence of air.

This idea of oxygen limitation as a colony grows is not unprecedented. Studies of *Bacillus cereus* showed that oxygen became undetectable at ~25 to 30 μM deep in colonies of this bacterium (38). Similarly, studies of a *Pseudomonas aeruginosa* biofilm showed that oxygen penetration was limited at a depth of ~50 to 90 μM (39, 40). In our studies, the *S*. Typhimurium anoxic core had to reach a minimum of ~200 μm in diameter before it supported robust aerobic growth of the colony (Fig. 8C).

### How and why does *S*. Typhimurium excrete Cbas?

#### How?

Even though we do not know how Cbas are excreted in *S*. Typhimurium, we do have some idea of what proteins may not be involved in Cba excretion. *S*. Typhimurium strains lacking two of the Btu proteins, namely, BtuC and BtuF, do not block Cba excretion across the inner membrane, as indicated by the large amounts of Cbas accumulated extracellularly in cultures of these strains (Fig. 11). Interestingly, we noticed that the absence of the BtuC or BtuF proteins had little impact on the uptake of CNCbl compared to the absence of BtuB, judging by the response of cultures as a function of CNCbl concentration in the medium (Fig. S5). Yet, cultures of strains lacking BtuC or BtuF accumulated higher extracellular levels of Cbas (Fig. 11). These observations agree with the idea that Cbas somehow cross the inner membrane and accumulate in the periplasm when BtuC or BtuF is absent, implying that the accumulation of Cbas in the periplasm somehow triggers their excretion. Notably, a Δ*btuB* strain accumulates very small levels of extracellular Cbas (Fig. 11). To explain this result, we tested the possibility that the BtuB protein could be involved in Cba excretion when a large amount of Cbas accumulated in the periplasm. However, chromosomal inactivation of *btuB* in a *btuC* strain did not result in a decreased level of extracellular Cbas (Fig. 11), suggesting that BtuB is likely not involved in the excretion of Cbas across the outer membrane.

#### Why?

Again, we do not know why *S*. Typhimurium excretes Cbas, but we suggest that Cba excretion might be triggered by problems caused by excessive Cba synthesis. This hypothesis is based on the observation that the amount of extracellular Cbas we measured increased as a function of the concentration of the precursors (CN)_2_Cbi supplemented in the medium (Table 1). It is possible that *S*. Typhimurium excretes Cbas when the intracellular Cba levels exceed the storage capacity of the cell. Alternatively, this bacterium may excrete Cbas because at very high levels, Cbas may interfere with as-yet unidentified metabolisms. Collectively, this work raises interesting questions regarding Cba metabolism and the role of this valuable cofactor in microbial community dynamics.

## MATERIALS AND METHODS

### Bacterial strains and chemicals

All strains constructed were derivatives of *Salmonella enterica* subsp. *enterica* serovar Typhimurium strain LT2 (referred to as *S*. Typhimurium). Genotypes of strains used in this study are listed in Table 2. Unless otherwise indicated, chemicals were obtained from Sigma-Aldrich and were used without further purification except for dicyanocobinamide [(CN)_2_Cbi], which was purified by HPLC to remove any trace of CNCbl. The method used to purify [(CN)_2_Cbi] is described below in RP-HPLC for Separation of Corrinoids.

**TABLE 2:** Bacterial strains and plasmids used in this study

Strain	Relative genotype	Source*^a^*
*E. coli* strains		
DH5∝	Φ80d*lac*ZDM15 *recA1 endA1 gyrA96 thi-1 hsdR17* (r_k_−, m_k_^+^) *supE44 relA1 deoR* Δ(*lacZYA-argF*) *U169 phoA*	Laboratory collection
*S.* Typhimurium LT2^*b*^ strains		
JE9426	*Salmonella enterica* subsp. *enterica* serovar Typhimurium LT2 reference genome	Gift from D.M. Downs
JE6583	Δ*araB9 metE205*	Kenneth Sanderson
JE7087	Δ*araB9* Δ*metE2701*::*cat*^+^	Lab collection
JE10079	Δ*araB9*	Lab collection
Derivatives of strain JE6583		
JE1299	*btuB7*::MudJ^*c*^	Lab collection
JE1626	*btuB7*::MudJ(*kan^+^*)/pKH3-3	Lab collection
JE28642	*btuB7*::MudJ(*kan^+^*)/pBR322	
JE16001	*btuC115*::*kan*^+^	Lab collection
JE28638	*btuC115*::*kan*^+^/pSeBTUC11	
JE28639	*btuC115*::*kan*^+^/pCV1	
JE28571	*btuF123*::*kan*^+^	Lab collection
JE28607	*btuF123*::*kan*^++^/pSeBTUF7	
JE28640	*btuF123*::*kan*^+^/pCV1	
JE28827	*btuC119 btuB7*::MudJ^*c*^	
JE1736	*cobU66*:: MudJ(*kan^+^*)	Lab collection
JE7089	Δ*metH2703*::*cat*^+^	Lab collection
JE8214	Δ*cobS1312*::*cat*^+^	Lab collection
JE8248	Δ*cobS1313*	Lab collection
JE15888	Δ*cobS1313*/pCOBS69	Lab collection
JE16852	Δ*cobS1313*/pBAD24	Lab collection
JE27701	*cobU66*::MudJ *metN2703 yjeH208 leuE3301*	
JE28897	*cobU66*::MudJ *metN2703 yjeH208 leuE3301/*pCOBUST1	
JE28065	*cobU66*::MudJ *metN2703 yjeH208 leuE3301/*pCV1	
JE27702	Δ*metH2704 cobU66*::MudJ(*kan^+^*) *metN2703 yjeH208 leuE330*	
JE28984	Δ*metH2704 cobU66*::MudJ(*kan^+^*) *metN2703 yjeH208 leuE3301/*pMETH2	
JE28985	Δ*metH2704 cobU66*::MudJ(*kan^+^*) *metN2703 yjeH208 leuE3301/*pCV1	
JE28303	Δ*acs2* Δ*pta127 cobU66*::MudJ(*kan^+^*)	
JE28339	*btuB8*::Tn*10*d(*tet^+^*) *cobU66*::MudJ(*kan^+^*)	
JE28428	Δ*acs2* Δ*pta127 cobU66*::MudJ(*kan^+^*) *btuB8*::Tn*10*d(*tet^+^*)	
JE25837	Δ*cbi241*::*cat^+^*^*d*^ (Δ*cbiA-cobS*::cat+)	
JE25838	*btuB7*::MudJ^*c*^ Δ*cbi241*::*cat+* (Δ*cbiA-cobS*::cat+)	
JE25839	*btuC115*::*kan*^+^ Δ*cbi241*::*cat+* (Δ*cbiA-cobS*::cat+)	
JE25840	*btuF123*::*kan*^+^ Δ*cbi241*::*cat+* (Δ*cbiA-cobS*::cat+)	
Derivatives of strain *JE7087*		
JE18413	Δ*thiC1137::kan*^+^	Lab collection
JE7088	Δ*metE2702*	Lab collection
JE10154	Δ*metE2702/*pKD46	Lab collection
JE25985	Δ*metE2702* Δ*cobB1375* Δ*cbi241*::*cat+* (Δ*cbiA-cobS*::cat+)	
Derivatives of strain *JE10079*		
JE19582	Δ*metN2703*::*cat*^+^	Lab collection
JE27521	Δ*cobS1312*::*cat*^+^	
Markers from *S.* Typhimurium 14028s strains from the BEI collection transduced into strain JE6583, resulting in the following strains		
JE27572	Δ*yjeH*::*cat^+e^*	
JE27573	Δ*leuE*::*cat^+f^* (aka *yeaS*)	
Plasmid	Description	Source
pKH3-3	*S*. Typhimurium LT2 *btuB ^+^* cloned into pBR322	(41)
pCV1	Complementation vector P*_araBAD_ bla+*	(42)
pSeBTUC11	*S*. Typhimurium LT2 *btuC*^+^ cloned into pCV1	
pSeBTUF7	*S*. Typhimurium LT2 *btuF*^+^ cloned into pCV1	
pCOBS69	*S*. Typhimurium LT2 *cobS*^+^ cloned into pBAD24	Lab collection
pCOBUST1	*S*. Typhimurium LT2 *cobUST*^+^ cloned into pCV1	
pMETH2	*S*. Typhimurium LT2 *metH*^+^ cloned into pCV1	
pKD3	Template plasmid carrying *cat* + gene	(43)
pKD46	Expresses the λ Red recombinase system	(43)

^*a*^ Unless otherwise stated, strains and plasmids were constructed during this work.

^*b*^ *S*. Typhimurium is the abbreviation of *Salmonella enterica* subspecies *enterica* serovar Typhimurium strain LT2.

^*c*^ MudJ is the abbreviation of MudII1734 (44).

^*d*^ Allele Δ*cbi241* represents a deletion starting 591 bp upstream of the *cbiA* gene and ending 360 bp into the coding sequence of *cobS*.

^*e*^ The genetic marker was obtained from the BEI strain collection (SGD_047/048_Cm F09), NIAID, NIH: *Salmonella enterica* subsp. *enterica*, Strain 14028s (Serovar Typhimurium) Single-Gene Deletion Mutant Library (45).

^*f*^ The genetic marker was obtained from the BEI strain collection (SGD_047/048_Cm F09), NIAID, NIH: *Salmonella enterica *subsp. *enterica*, Strain 14028s (Serovar Typhimurium) Single-Gene Deletion Mutant Library (45).

### Culture media and growth studies

All strains were grown in LB (Difco), nutrient broth (NB, Difco; supplemented with NaCl [5 g/L]), or no-carbon essential (NCE) minimal medium (46). NCE minimal medium was supplemented with MgSO_4_ (1 mM) and Wolfe’s trace minerals (1×) (47). Ribose (22 mM), glycerol (22 mM), or ethanolamine (90 mM) was added to the minimal medium as a carbon and energy source when cultures were grown aerobically. When ethanolamine was used as the sole source of carbon and energy, the medium was supplemented with methionine (300 μM). Ribose (50 mM) was used as the carbon and energy source for anaerobic liquid cultures. When used, antibiotics were added at the following concentrations: ampicillin, 100 μg/mL; chloramphenicol, 20 μg/mL; and kanamycin, 50 μg/mL. For growth analysis, starter cultures were grown overnight (~20 h) at 37°C in LB or NB, and cultures (2%, vol/vol) were used to inoculate 196 μL of fresh medium placed in each well of a 96-well microtiter plate. Microtiter plates (Fisher Scientific) were incubated at 37°C inside the temperature-controlled chamber of a microtiter plate reader (BioTek Instruments), and plates were continuously shaken using the medium setting of the instrument. Cell density was monitored at 630 nm, and data were analyzed using the Prism software package (v10) (GraphPad). Cultures for anaerobic growth assays were prepared inside an anoxic Coy chamber using Balch tubes (30 mL) or Balch bottles (500 mL) (47) that were sealed and incubated at 37°C with shaking at 180 rpm in an Innova 43 shaking incubator.

#### Liquid co-culture assays

Starter cultures were grown in LB (1 mL) overnight (~20 h) at 37°C. To minimize thiamine carryover from the overnight culture, cultures were grown in NB when strain JE18413 (*araB metE thiC*) was used in a co-culture. NCE minimal medium was inoculated with overnight cultures (1%, vol/vol, for cultures grown on glycerol; 2%, vol/vol, for cultures grown on ethanolamine). Glycerol (22 mM) or ethanolamine (90 mM) was used as the source of carbon and energy. NCE minimal medium was supplemented with (CN)_2_Cbi (10 and 200 nM, when glycerol and ethanolamine were used as carbon and energy sources, respectively) and DMB (300 mM). When ethanolamine was used as the source of carbon and energy, the medium was supplemented with L-Met (300 μM). Antibiotic resistance was used to differentiate CFUs from each strain used in a co-culture. Ten-fold serial dilutions were made using sodium chloride solutions (NaCl, 0.9%, wt/vol), and samples were plated on LB agar (1.5%, wt/vol, Difco) with proper antibiotics. Plates were incubated overnight (~20 h) at 37°C before colonies were counted. Co-cultures grown in glycerol were set up in 5 mL cultures in tubes and were incubated in an Innova 43 shaking incubator (180 rpm). Co-cultures grown on ethanolamine were grown in 200 mL cultures in microtiter plates (Fisher Scientific) and were incubated at 37°C inside the temperature-controlled chamber of a microtiter plate reader (BioTek Instruments). For each Cba auxotrophic strain grown in co-culture in a medium containing ethanolamine, a single-strain culture control was grown with CNCbl (200 nM). Samples were taken from co-cultures to determine CFUs per milliliter once the single-strain culture of the auxotrophic strain reached an early stationary phase.

#### Co-culturing on solid medium

Starter cultures for both the Cba producer and the Cba auxotroph were grown in LB (1 mL) overnight (~20 h) at 37°C. Cba auxotrophic cells were harvested by centrifugation at 6,000 × *g* for 10 min and washed twice with a NaCl solution (0.9%, wt/vol), then resuspended to a final volume of 1 mL of the same NaCl solution. Samples from washed cultures (100 mL, ~10^7^ cells) were mixed with molten NCE noble agar (25 mL, 1.5%, wt/vol; Thermo Scientific) supplemented with glycerol (22 mM), CN_2_Cbi (10 nM), DMB (300 μM), and X-gal (40 mg/mL). Plates were allowed to solidify at room temperature for 30 min. Ten-fold dilutions were prepared from the overnight culture of the Cba producer using a NaCl solution (0.9%, wt/vol), and samples (100 mL) were spread from a 10^−6^ dilution onto the plates. Plates were incubated at 37°C for 24–48 h.

### Plasmid construction

All plasmids used in this work are listed in Table 2. Primers used in this study were synthesized by Integrated DNA Technologies, Inc. (Coralville, IA) and are listed in Table 3. Genes *btuC* (*STM1340*), *btuF* (*STM0206*), *metH* (*STM4188*), and *cobUST* (*STM2018-STM2016*) were amplified using strain JE9426 gDNA as the template and Phusion High-Fidelity Polymerase (Thermo Fisher Scientific). PCR products were analyzed on 0.8% agarose (wt/vol, Apex BioResearch) gels after staining with ethidium bromide (Sigma, 0.5 mg/mL) and cleaned up using the Wizard SV Gel and PCR Cleanup System (Promega). PCR products were cloned into plasmid pCV1 followed by BspQI restriction digestion, as described elsewhere (42, 48). Resulting plasmids were transformed into *Escherichia coli* DH5α cells, followed by plating on media supplemented with appropriate antibiotics; colony PCR was performed to identify plasmids carrying an insert with the expected size. Plasmids were isolated using the Wizard Plus SV Miniprep Kit (Promega), and the nucleotide sequence of the cloned region was verified by Eton Bioscience, Inc. Constructed plasmids were transformed into *S*. Typhimurium strains by electroporation, as described elsewhere (49).

**TABLE 3:** List of primers used in this study

Primer^*a*^		Sequence
Primers used for cloning		
788.btuC.BspQI_F		NNGCTCTTCNTTCatgctgacttttgcccg
789.btuC.BspQI_R		NNGCTCTTCNTTActaacgcgcggatttgag
574.btuF.BspQI_R		NNGCTCTTCNTTAggctctaattcacctgtg
575.btuF.BspQI_F		NNGCTCTTCNTTCatggctaagcaaatgttc
44.BSPQ_cobT_R		NNGCTCTTCNTTATTATGTTGCGTTTGCGTTCCCC
46.BSPQ_cobUS_F		NNGCTCTTCNTTCATGATGATTCTGGTGACGGGCG
602.metH.BspQI_F		NNGCTCTTCNTTCatgtctcatgttgcccg
603.metH.BspQI_R		NNGCTCTTCNTTAtcagtccgcatcgtaacc
Primers used for chromosomal deletions		
32.W_CbiP-CobS_R		ACCAGAATTTTTGCCAGTAGCACAAAAATAAGCGCCCATATGAATATCCTCCTTAG
50. W_PcbiA_F		AATGAGAATTCTCATCCTCCATCGTTATGAATAGCAGTGTAGGCTGGAGCTGCTTC

^*a*^ All primers were synthesized by Integrated DNA Technologies, Inc. (Coralville, IA).

### Construction of chromosomal deletions

Chromosomal deletion of the cobamide biosynthetic operon (*cbiA-cobS*) was constructed using the phage λ Red recombinase system described elsewhere (43). Briefly, an amplicon containing the chloramphenicol resistance cassette with flanking homologous regions to the cobamide biosynthetic operon (*cbiA-cobS*) was amplified, and an amplicon (~1 μg) was electroporated into the electrocompetent strain harboring plasmid pKD46 (JE10154), which encoded the λ Red recombinase. Cells were then recovered with 1 mL of super optimal broth supplemented with glucose (20 mM) and plated on LB agar (1.5%, wt/vol) supplemented with chloramphenicol (20 μg/mL). Successful recombination of chloramphenicol-resistant colonies was verified by a colony PCR. Primers used for chromosomal deletion are listed in Table 3.

### Strain constructions

Strains used in these studies were constructed using a high-frequency general-transducing bacteriophage P22 strain (50) using protocols described elsewhere (49). Where necessary, colony PCR was performed targeting the gene of interest to verify the presence of transduced markers using GoTaq Green Master Mix (Promega) as per the manufacturer's instructions. Donor strains for null alleles of *leuE* and *yjeH* were obtained from a collection of targeted single-gene deletions in *Salmonella enterica* sv. Typhimurium 14028s (45).

### Harvesting extracellular corrinoids

To harvest extracellular corrinoids, cultures were centrifuged at 6,000 × *g* for 15 min at 4°C. Cell pellets were stored at −80°C and used to determine intracellular corrinoid content. Supernatants were filtered through a 0.22 μM PES filter (Avantor VWR) to remove remaining cells. For partial purification of corrinoids, a SepPak C18 Plus cartridge (360 mg sorbent per cartridge, 55–105 mm particle size; Waters) was activated with methanol (10 mL) and water (10 mL). The supernatant was passed through the cartridge, and unbound impurities were washed with water (20 mL). Corrinoids were eluted with methanol (1.5 mL) and dried overnight using a VacFuge Plus instrument (Eppendorf). Dried corrinoids were resuspended in sterile water and stored at 4°C until further use.

### Extraction of intracellular corrinoids

Isolation of intracellular corrinoids was performed as described elsewhere (51). Briefly, the frozen cell pellet was resuspended in buffer A (10 mL, 50 mM KH_2_PO_4_ [pH 6.5], 5 mM KCN) and methanol (20 mL). Cell lysis and corrinoid extraction were performed at 65°C for 2 h with shaking (220 rpm). Cell debris was removed by centrifugation at 43,000 × *g* for 1 h in an Avanti JP20-XVI centrifuge equipped with a JA-25.50 rotor. Corrinoids in the supernatant were partially purified using a SepPak C18 Plus cartridge as described in Harvesting Extracellular Corrinoids.

### RP-HPLC was used to resolve intracellular and extracellular corrinoids

To separate extracellular and intracellular corrinoids, we followed a protocol described elsewhere (52). Briefly, RP-HPLC was performed on a Shimadzu Prominence Ultra-Fast Liquid Chromatography SPD-M30A instrument equipped with a Synergi 4 µm Hydro-RP 80 Å 150 × 4.6 mm column. Potassium cyanide was added to a final concentration of 25 mM to resuspend corrinoids, followed by incubation at 80°C for 10 min. Corrinoids were passed over a Spin-X centrifuge tube filter (Corning, 0.45 μm cellulose acetate) for 5 min at 13,000 × *g* prior to RP-HPLC. The column was equilibrated with 80% buffer C (KH_2_PO_4_ [0.1 M] [pH 6.5] and KCN [10 mM]) and 20% buffer B (KH_2_PO_4_ [0.1 M] [pH 8] and KCN [10 mM] + acetonitrile at a 1:1 ratio) for 2.5 min at a flow rate of 1.0 mL/min. The percentage of buffer B was developed using a linear gradient to 35% over 9 min, then further increased to 100% buffer B over 7 min, and for an additional 5 min, the column was equilibrated with 100% buffer B. Corrinoids were detected by measuring the absorbance at 367 nm, and fractions containing corrinoids were collected. Fractions were desalted and resuspended in sterile water using a SepPak C18 Plus cartridge, as described under Harvesting Extracellular Corrinoids.

### Bioassay for detection and quantification of Cbas

For detection and quantification of extracellular Cbas, we used growth of strain JE8214 (*metE cobS*) in a liquid medium. We used the growth rate of the bioassay strain as an indirect measurement of Cba present in the growth medium. Overnight cultures (~20 h) of strain JE8214 were grown in NB (1 mL). Samples from the overnight culture (2%, vol/vol) were used to inoculate 196 μL of fresh NCE medium supplemented with glycerol (22 mM) and varying concentrations of CNCbl placed in each well of a 96-well microtiter plate. Microtiter plates (Fisher Scientific) were incubated at 37°C inside the temperature-controlled chamber of a microtiter plate reader (BioTek Instruments) for 48 h, and plates were continuously shaken using the medium setting of the instrument. Cell density was monitored via optical density at 630 nm, and data were analyzed using Prism software package (v10, GraphPad), and growth rates were calculated using an online software (https://scott-h-saunders.shinyapps.io/gompertz_fitting_0v2/). Growth rate was plotted against the provided CNCbl concentration, and the linear range of the curve was used to determine the unknown Cba concentrations.

### Colony formation assays

To test the Cba-dependent visual growth of colonies under aerobic conditions, we used NCE plates supplemented with noble agar (Thermo Scientific, 1.6%, wt/vol), 1× Wolfe’s trace minerals (47), MgSO_4_ (1 mm), sorbitol (30 mM), 1,2-propanediol (10 mM), and CoCl_2_ (4 mM). Ten-fold dilutions were prepared from an overnight culture (20 h) grown in LB (1 mL) using a NaCl solution (0.9%, wt/vol), and samples (100–200 μL) were spread from 10^−6^ dilution on to the plates. Plates were transferred into an anaerobic jar that contained a GasPak EZ anaerobe container system sachet (Becton Dickinson) and incubated under anoxic conditions at 37°C for an indicated period. Upon anoxic growth, plates were transferred to aerobic conditions and further incubated at 37°C. For measurements of microcolonies, an Olympus SZ61 microscope and Cell Sens Standard (v1.2) software were used.

#### Colony formation on nitrocellulose membrane

NCE agar plates were prepared supplemented with noble agar (Thermo Scientific, 1.6%, wt/vol), 1× Wolfe’s trace minerals (47), MgSO_4_ (1 mM) sorbitol (30 mM), 1,2-propanediol (10 mM), CoCl_2_ (4 μM), l-(+)-arabinose (250 mM), and CNCbl (50 pM). Sterilized nitrocellulose membranes (82 mm, 0.45 μm; BioRad) were placed on the surface of agar plates and allowed to dry for 5 min at room temperature. Strains were grown in LB (1 mL) overnight (20 h) at 37°C. Ten-fold dilutions were prepared from an overnight culture (20 h) grown in LB using a NaCl solution (0.9%, wt/vol), and samples (75 μL) were spread from 10^−6^ dilution onto the plates using glass beads. Plates were incubated for 24 h at 37°C. After incubations, membranes were transferred onto a new set of NCE agar plates like the first set, except the new plates were devoid of CNCbl. Plates were further incubated for ~4 to 6 days at 37°C. A similar procedure was followed when colony formation was assayed with ethanolamine as the source of carbon, except NCE medium contained ethanolamine (90 mM), L-Met (300 μM), and potassium tetrathionate (40 mM).

## Data Availability

All the data generated by this work are contained in this paper and its supplemental material.

## SUPPLEMENTAL MATERIAL

Supplemental figures spectrum.01363-26-s0001.pdf Figures S1 to S6.

ASM does not own the copyrights to Supplemental Material that may be linked to, or accessed through, an article. The authors have granted ASM a non-exclusive, world-wide license to publish the Supplemental Material files. Please contact the corresponding author directly for reuse.
