# Supplementary material for: Cobamide excretion allows Salmonella to transition from its anaerobic to aerobic lifestyle
Source: Microbiol Spectr. 2026 Jul 24;14(9):e01363-26. doi: 10.1128/spectrum.01363-26 (PMC13532332; doi:10.1128/spectrum.01363-26)
Supplement: Supplementary file 1 [file spectrum.01363-26-s0001.pdf]

SUPPLEMENTARY MATERIAL

Cobamide excretion allows *Salmonella* to transition from its anaerobic to its aerobic lifestyle

Lahiru Malalasekara[1] and Jorge C. Escalante-Semerena[1]*
[1]Department of Microbiology, University of Georgia, Athens USA

*Corresponding author: 330G Cedar Street Building C, 136 Cedar Street, Athens, GA 30602 USA; T: +1 (706) 542-2651, F: +1 (706) 542-2815, Email: jcescala@uga.edu

Running title: Cba excretion by *Salmonella*

Keywords: corrinoid excretion, *Salmonella* metabolism, cobamide-dependent growth, *Salmonella* lifestyle transition

Supplementary Figures

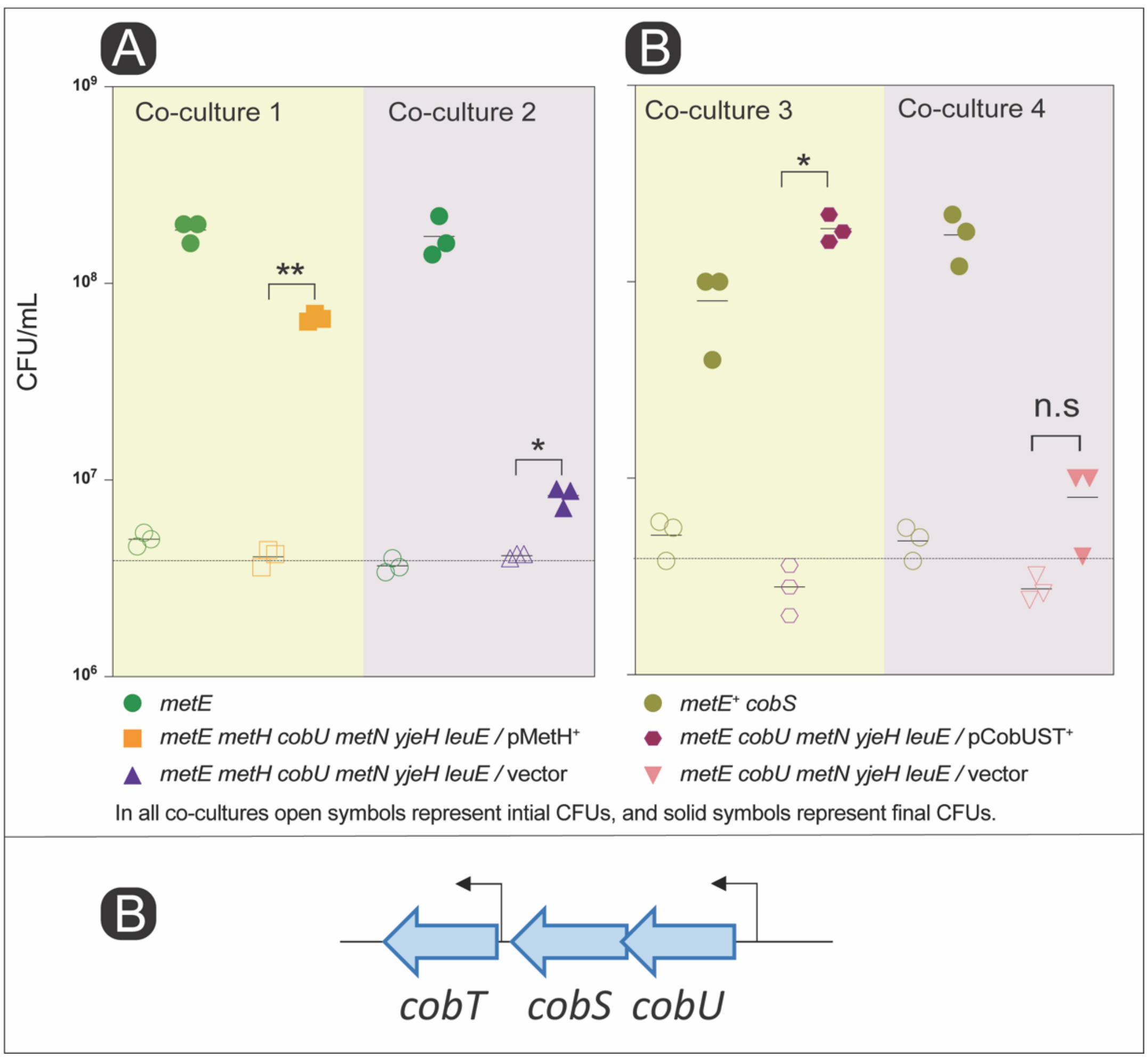

Figure S1. Genetic complementation to rescue the growth of a methionine auxotroph (A) and a Cba auxotroph (B). Four different co-cultures were grown (1-4) in NCE minimal medium (5 mL) supplemented with glycerol (22 mM), $(CN)_2$Cbi (10 nM) DMB (300 μM) and L-(+)-arabinose (500 μM).Viable cell counts of each strain were obtained using their unique antibiotic markers at two different time points of growth: initial and early stationary phase (after 8 h). The experiment was repeated two times with three technical replicates, with a representative result shown. A

paired Student's *t* test was performed to determine statistical significances (ns, not significant; *, *p*= 0.0198, **, *p*= 0.001). **C.** depicts the arrangement of *cobU* (546 bp) *cobS* (744 bp) and *cobT* (1071 bp) genes in the chromosome (spanning from 2099682 bp to 2097300 bp) of *S*. Typhimurium.

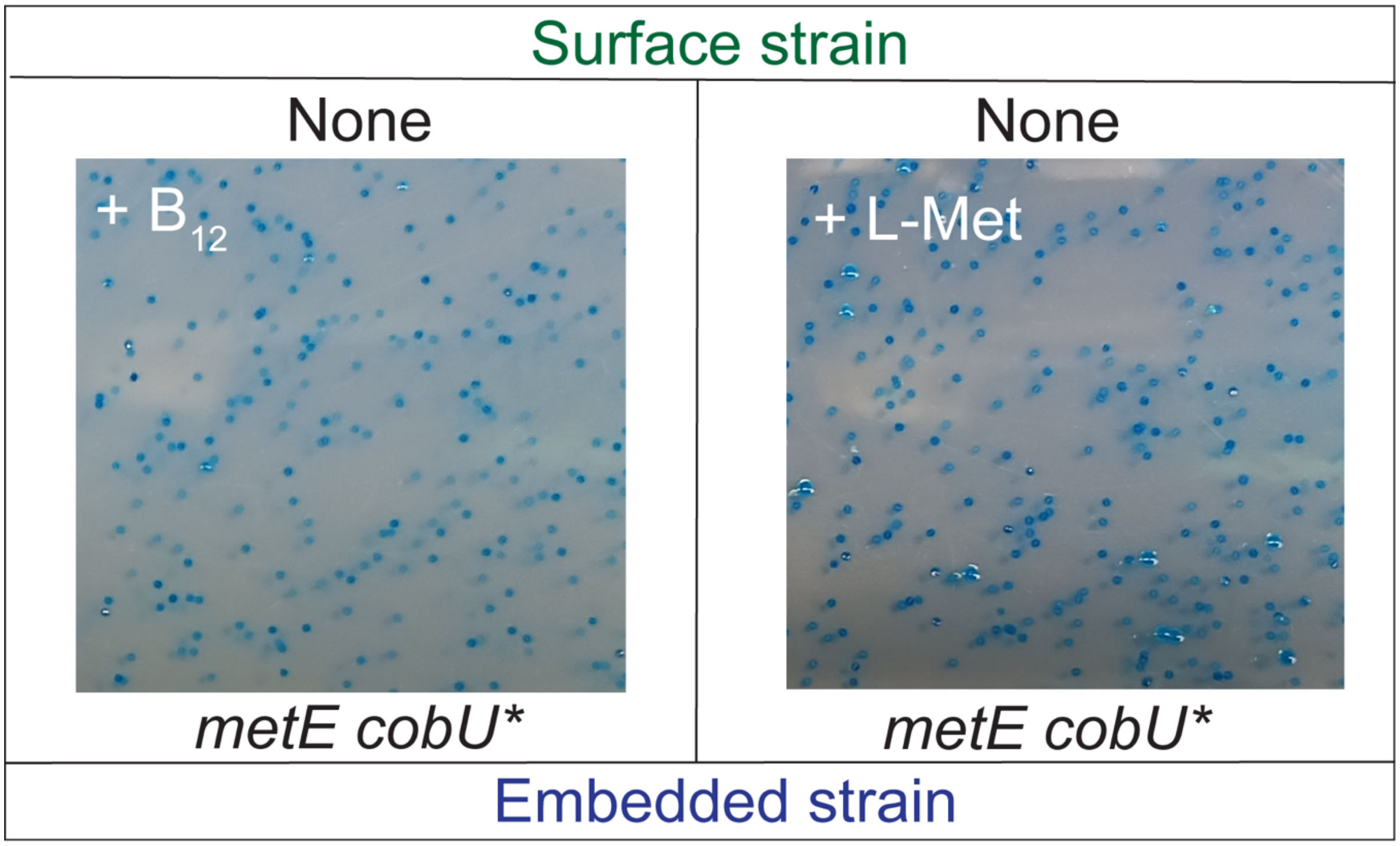

Figure S2. Blue color was observed upon the growth of the Cba auxotrophic reporter strain. Strain genotype describing a *cobU* mutation refers to a *cobU-lacZ*$^+$ transcriptional fusion (1). Cells (~ 1000-2000 CFU) from an overnight culture (20 h, in LB) of strain JE27701 (Δ*metE cobU-lacZ*) were embedded into NCE agar (25 mL, 1.5 % w/v) supplemented with glycerol (22 mM) and 5-bromo-4-chloro-3-indolyl-β-D-galacto-pyranoside (X-gal, 40 μg/mL). Medium was supplemented with either CNCbl (50 pM) or L-Met (500 μM) as indicated. Plates were incubated at 37°C for 48 h. The asterisk on the genotype of strain JE27701 indicates the presence of chromosomal null alleles of Δ*metN*, Δ*yjeH* and Δ*leuE* in the strains. Experiment was repeated three times with a representative result shown.

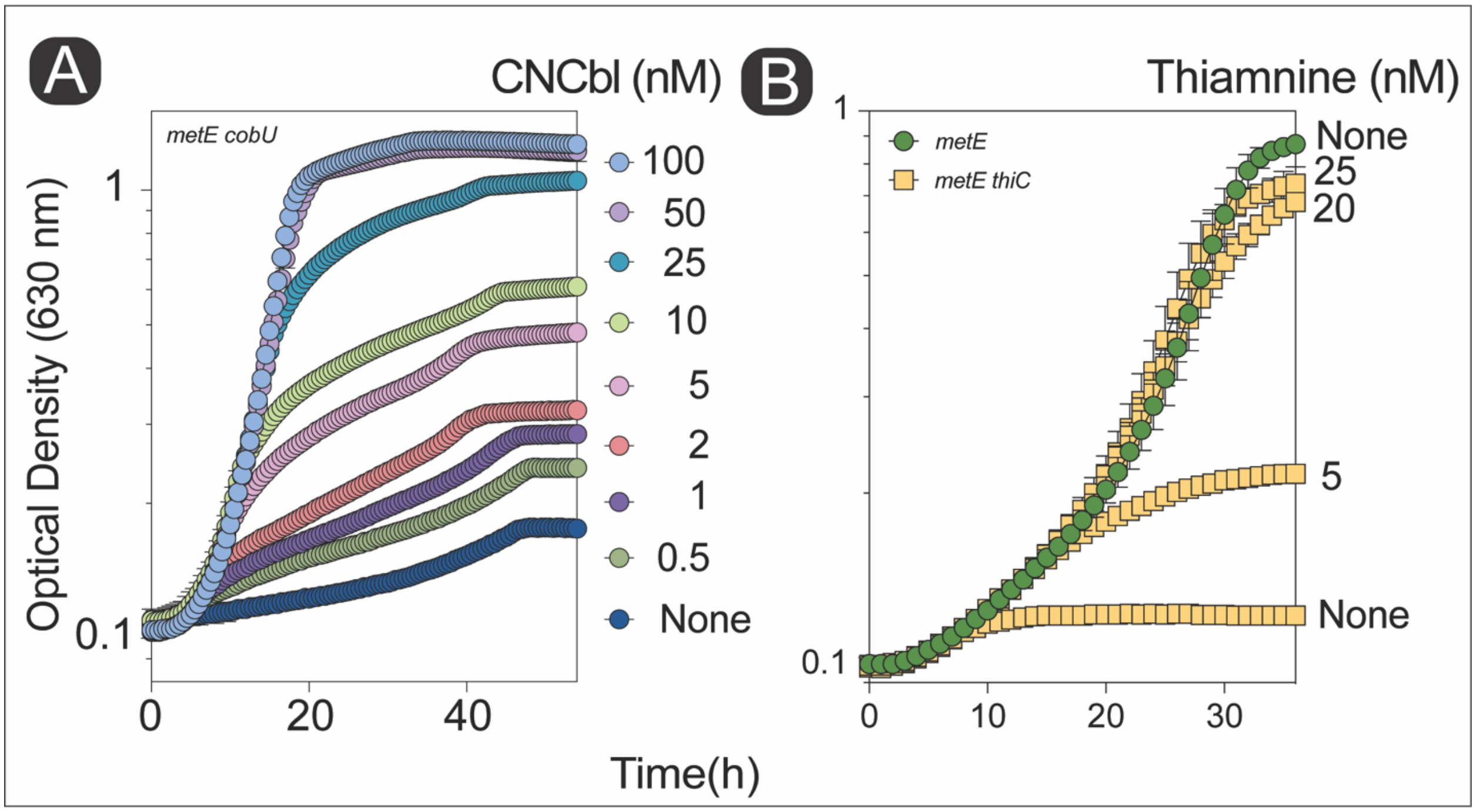

Figure S3. Minimum requirement of CNCbl and thiamine to restore the growth of a Cba auxotroph and a thiamine auxotroph respectively. Cultures were grown in NCE minimal medium supplemented with ethanolamine (90 mM) and L-methionine (300 μM). Strain JE1736 (metE cobU) (**A**) or JE18413 (*metE thiC*) (**B**) was supplemented with concentrations of CNCbl (**A**) or thiamine.hydrochloride (**B**) as indicated. Each experiment was repeated two times with technical triplicates. Representative graphs are shown, with error bars indicating standard deviations.

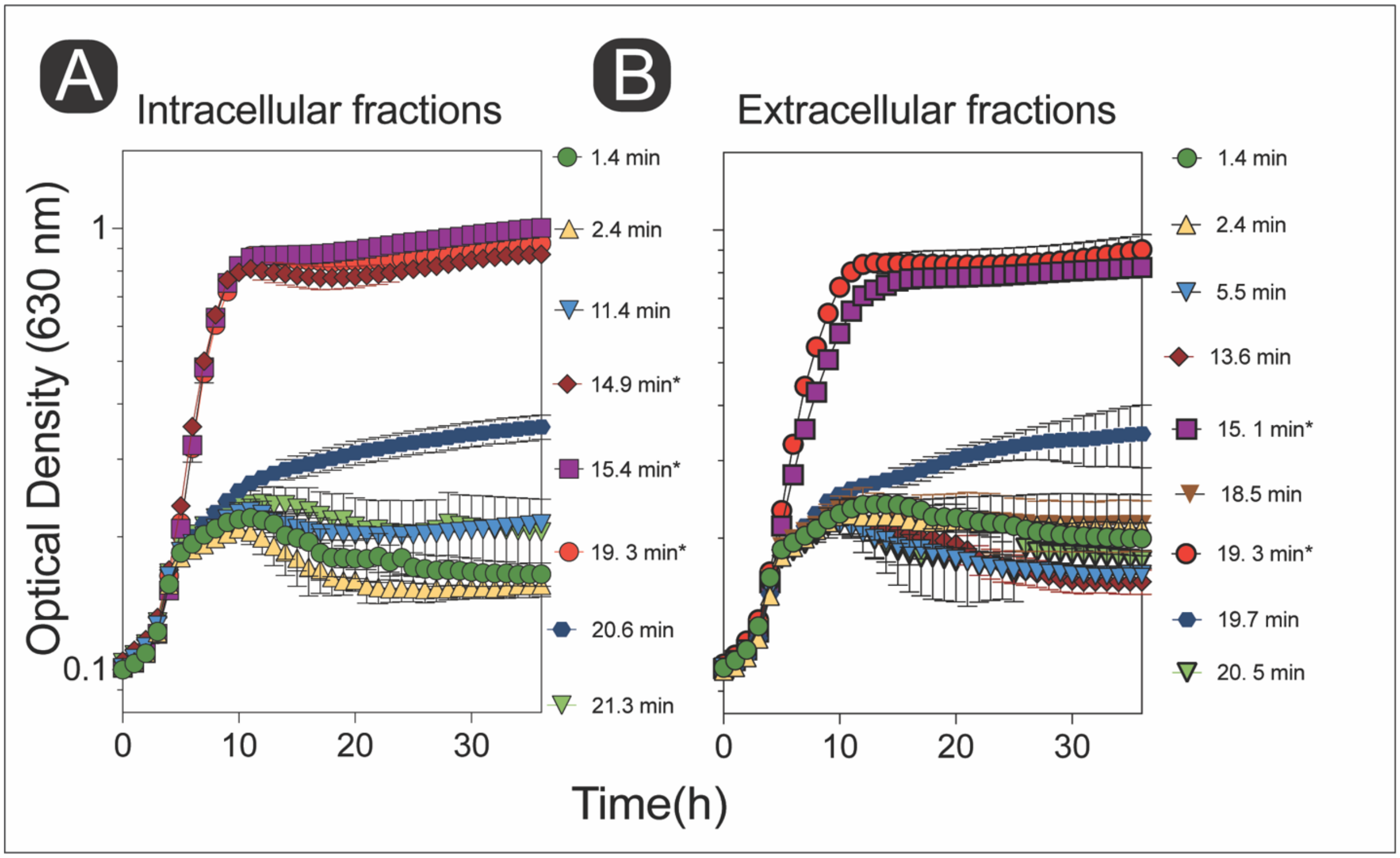

Figure S4. Bioassay for detection of Cbas in RP-HPLC purified fractions. Intracellular **(A)** and (**B**) extracellular corrinoids were extracted and separated from a culture (250 mL) of strain JE10079 grown anaerobically as described under Materials *& Methods* and Fig 7. Fractions collected at indicated timepoints were purified using a SepPak C18 cartridge (Waters) and inoculated to a culture of strain JE8214 grown in NCE glycerol (22 mM) medium at ~10X final concentration. Each experiment was repeated three times with technical triplicates. Representative graphs are shown, with error bars indicating standard deviations.

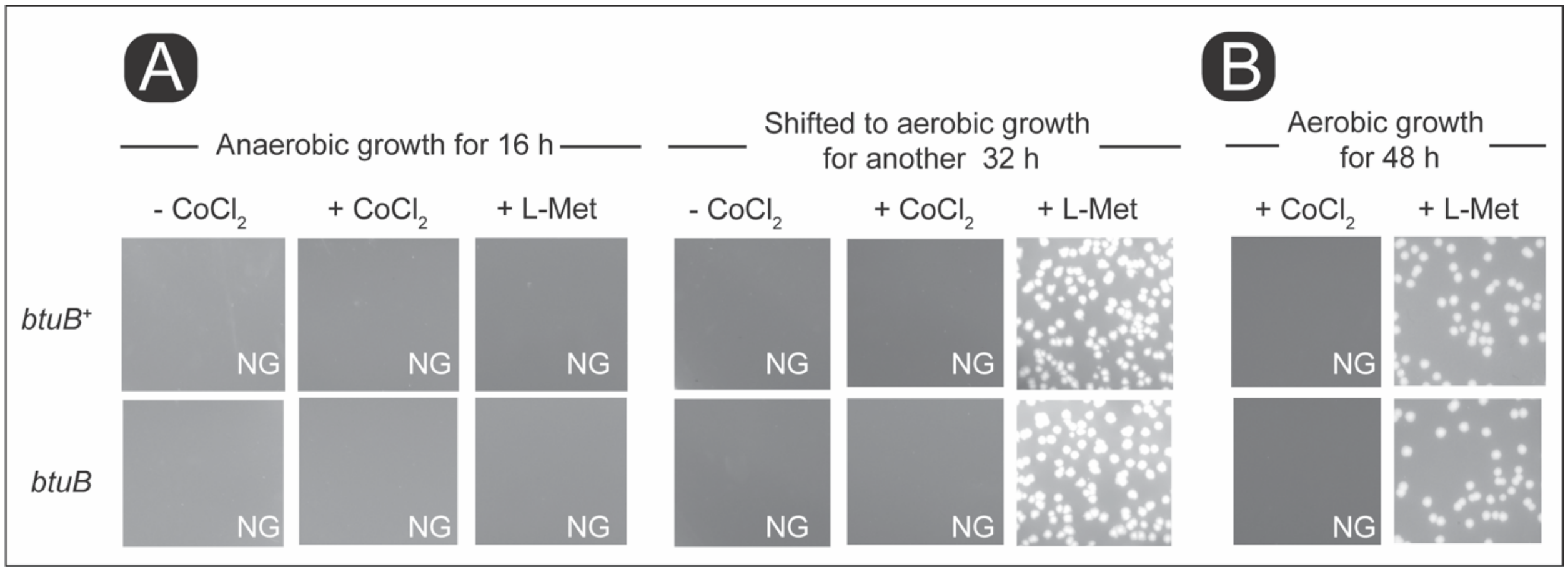

Figure S5. Minimal growth requirements for Cba dependent aerobic colony growth. Cells of strains JE6583 (*metE*), and JE1299 (*metE btuB*) were spread on NCE agar plates supplemented sorbitol (30 mM) and 1,2-propandiol (10 mM). L-methionine (L-Met, 300 μM) or $CoCl_2$ (4 μM) was added to the medium as indicated. **A**. Plates were incubated for 16 h under anoxic conditions and further incubated for another 32 h after shifting into aerobic conditions. **B** Plates were incubated for 48 h aerobically. **NG**, no visible growth.

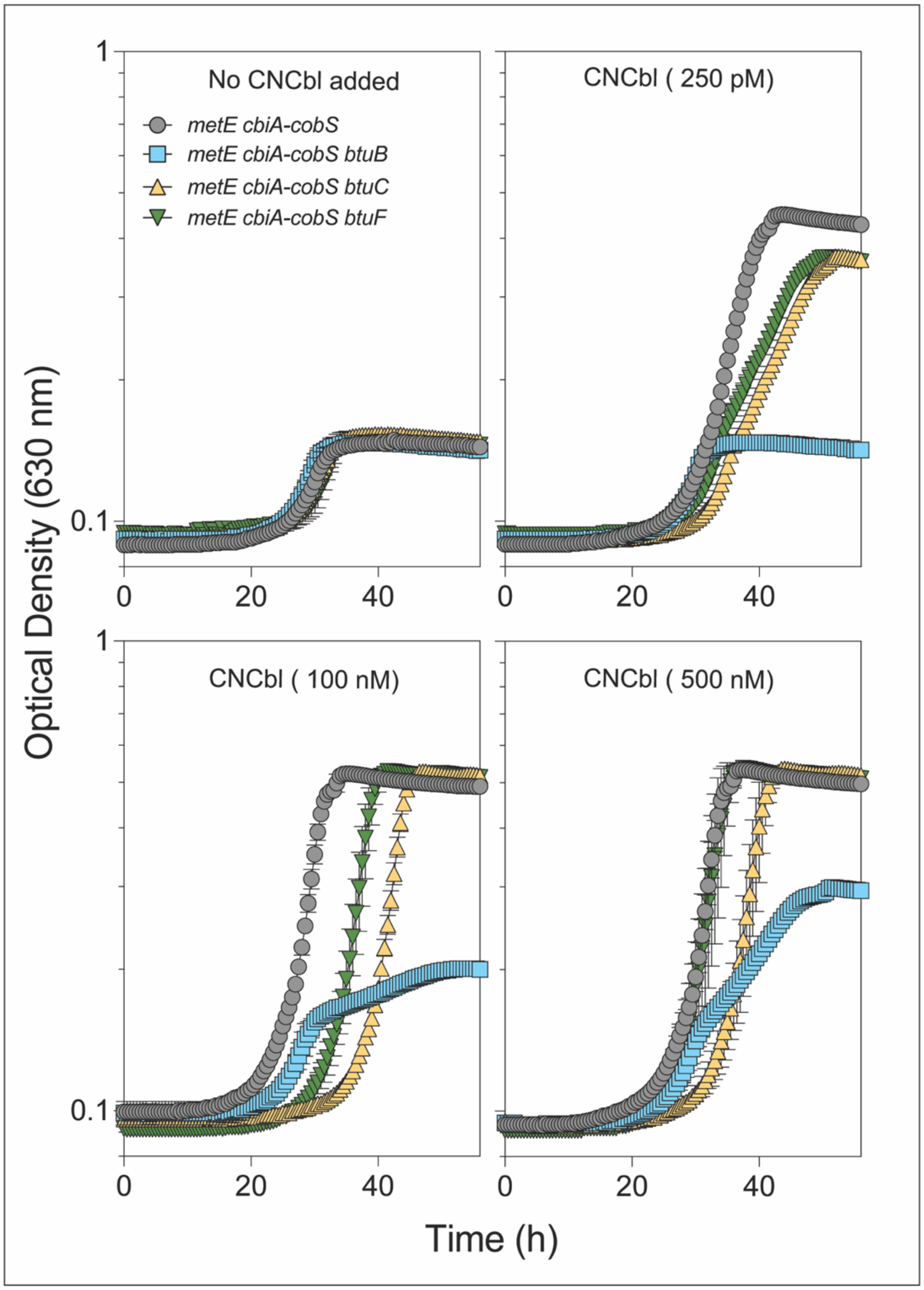

Figure S6. Sensitivity of corrinoid transporters in *Salmonella*. Cultures were grown anaerobically in NCE minimal medium provided with Ribose (50 mM), 1,2-PDL (10 mM), $CoCl_2$ (4 μM) and supplemented with different concentrations of CNCbl as indicated. Each experiment was repeated three times with technical triplicates. Representative graphs are shown, with error bars indicating standard deviations. Strains used for this experiment were JE28837 (*metE cbiA-cobS*), JE28838 (*metE cbiA-cobS btuB*), JE28839 (*metE cbiA-cobS btuC*) and JE28840 (*metE cbiA-cobS btuF*). All strains carried a null allele of *araB*.

References

1. Escalante-Semerena JC, Roth JR. 1987. Regulation of cobalamin biosynthetic operons in *Salmonella typhimurium*. J Bacteriol 169:2251–2258.
